# Childhood trauma and brain structure in children and adolescents

**DOI:** 10.1016/j.dcn.2022.101180

**Published:** 2022-11-23

**Authors:** Matthew Peverill, Maya L. Rosen, Lucy A. Lurie, Kelly A. Sambrook, Margaret A. Sheridan, Katie A. McLaughlin

**Affiliations:** a University of Wisconsin, Department of Psychiatry, 6001 Research Park Blvd., Madison, WI 53719, USA; b Harvard University, Department of Psychology, William James Hall, 10th Floor, 33 Kirkland St., Cambridge, MA 02138, USA; c Smith College, Program in Neuroscience, Clark Science Center, 44 College Ln, Northampton, MA 01063, USA; d University of North Carolina, Chapel Hill, Department of Psychology, 235 E Cameron Ave., NC 27599, USA

**Keywords:** Threat, Deprivation, Adolescence, Cortical thickness, Brain structure

## Abstract

The dimensional model of adversity proposes that experiences of threat and deprivation have distinct neurodevelopmental consequences. We examined these dimensions, separately and jointly, with brain structure in a sample of 149 youth aged 8–17—half recruited based on exposure to threat-related experiences. We predicted that greater threat would be uniquely associated with reduced cortical thickness and surface area in brain regions associated with salience processing including ventromedial prefrontal cortex (vmPFC), anterior cingulate cortex (ACC), and insula, and that deprivation experiences would be uniquely associated with reductions in cortical thickness and surface area in frontoparietal areas associated with cognitive control. As predicted, greater threat was associated with thinner cortex in a network including areas involved in salience processing (anterior insula, vmPFC), and smaller amygdala volume (particularly in younger participants), after controlling for deprivation. Contrary to our hypotheses, threat was also associated with thinning in the frontoparietal control network. However, these associations were reduced following control for deprivation. No associations were found between deprivation and brain structure. This examination of deprivation and threat concurrently in the same sample provided further evidence that threat-related experiences influence the structure of the developing brain independent of deprivation.

## Highlights

• Threat-related experiences were associated with thinner cortex in multiple areas.
• Threat-related thinning spanned salience, default, and somatomotor networks.
• This thinning was robust to control for deprivation experiences.
• Younger, threat-exposed participants had smaller amygdala volumes.

## 1 Introduction

Increasing evidence suggests that adverse childhood experiences are associated with numerous aspects of neural development, including brain structure (Hart and Rubia, 2012; McLaughlin, Weissman et al., 2019; Teicher et al., 2016). In particular, experiences of adversity have been associated with reduced cortical thickness and surface area, as well as reduced volume of sub-cortical structures including the amygdala and hippocampus in children and adolescents (Colich et al., 2020, Gold et al., 2016, Kelly et al., 2015, Lim et al., 2014, McLaughlin et al., 2016, McLaughlin et al., 2019). We have proposed that threat and deprivation are dissociable dimensions of adverse environmental experiences that have neurodevelopmental consequences which are at least partially distinct (McLaughlin et al., 2014, Sheridan and McLaughlin, 2014). However, prior studies of childhood adversity have been limited in their ability to jointly examine these dimensions of experience in relation to brain structure. In the current study, we separately test the associations of continuous measures of threat and deprivation with brain structure in a large sample of youth with a broad variety of adversity experiences.

The dimensional model of adversity suggests that adverse childhood experiences reflect underlying dimensions of environmental experience that are shared by numerous types of adversity. In particular, this model distinguishes between two central dimensions of adverse environmental experiences: threat and deprivation. Threat is defined as experiences that involve harm or threat of harm to the physical integrity of the child and includes experiences involving interpersonal violence such as physical abuse, sexual abuse, witnessing domestic violence, or encountering community violence. Deprivation is defined as a reduction in expected social and cognitive inputs from the environment and includes experiences such as physical neglect, emotional neglect, institutional rearing, parental separation, and lack of cognitive stimulation. The dimensional model improves upon single-exposure and cumulative risk approaches by accounting for the co-occurrence of adverse experiences without assuming that all experiences influence neurodevelopment through the same underlying mechanisms (See McLaughlin et al., 2014, McLaughlin et al., 2021; Sheridan and McLaughlin, 2014 for review).

The dimensional model posits that experiences of threat will have the largest associations with brain systems involved in emotional processing and the detection of salience and threat (McLaughlin et al., 2014, McLaughlin et al., 2019). Extensive evidence shows that threatening experiences in childhood are associated with shifts in information processing that facilitate the rapid identification of threat, including greater perceptual sensitivity and attention biases to threat along with hostile attribution biases (Dodge et al., 1995, Pollak et al., 2000, Pollak and Sinha, 2002). Children who have experienced violence also exhibit heightened emotional reactivity to threat cues (Heleniak et al., 2016, Jenness et al., 2021, McCrory et al., 2011), altered fear learning (DeCross et al., 2022, Machlin et al., 2019, McLaughlin et al., 2016), and changes in learning and memory in the presence of threat cues (Lambert et al., 2019, Lambert et al., 2017). Importantly, these changes are likely adaptive for young people in threatening environments such that they may help them mobilize defensive responses to promote safety. However, these changes may result in over-identification of threat in safe contexts, leading to over-generalization of fear responses and psychopathology (Keding and Herringa, 2016, Marusak et al., 2015, Pine et al., 2005). We would thus expect that experiences of threat would be associated with changes in brain systems involved in processing salience and threat cues. This includes the salience network—encompassing the anterior insula, dorsal anterior cingulate, and the amygdala, and a network referred to in cortical network discovery studies as the limbic network (e.g., Yeo et al., 2011)—including the lateral and medial orbitofrontal cortex (OFC), ventromedial prefrontal cortex (vmPFC), and temporal pole. Indeed, previous studies focused on the impact of child abuse—an experience involving a high degree of threat—on brain structure have found evidence of reduced volume and/or thickness in salience processing regions including medial OFC, vmPFC, and temporal pole as well as reduced amygdala and hippocampus volume (Colich et al., 2020, Gold et al., 2016, Kelly et al., 2015, Lim et al., 2014, McLaughlin et al., 2016). A recent systematic review of over 100 studies of childhood adversity and neural development confirmed that exposure to threat, but not deprivation, was associated with reduced volume of amygdala and vmPFC as well as heightened activity in the salience network in response to negative emotional stimuli (McLaughlin et al., 2019).

In contrast, experiences of deprivation—such as neglect, institutional rearing, and low levels of cognitive stimulation—are associated with reduced performance on tasks of attention and executive function (Finn et al., 2017, Sheridan et al., 2012, Spielberg et al., 2015). Correspondingly, the dimensional model posits that the frontoparietal control network, which supports executive function (Corbetta and Shulman, 2002, Curtis and D’Esposito, 2003) may be particularly impacted by experiences of deprivation. Indeed, prior studies on adverse experience characterized by deprivation, including institutional rearing (Herzberg et al., 2018, Hodel et al., 2015, McLaughlin et al., 2014), neglect (physical and emotional; Edmiston et al., 2011), and low cognitive stimulation (Rosen et al., 2018) have found evidence of reduced cortical thickness and volume in lateral prefrontal and parietal cortex (see McLaughlin et al., 2019 for review).

Previous studies have demonstrated that threat and deprivation are separately associated with brain structure differences in distinct neural systems. However, prior work has predominantly recruited samples of children based on exposure to either threat or deprivation without assessing or controlling for the other. As argued elsewhere (McLaughlin et al., 2021) the strongest evidence for distinct influences of these dimensions of adversity on neural development would come from documentation of associations of one dimension of adversity with brain structure while controlling for the other dimension of adversity. Here, in a large sample of children and adolescents aged 8–16 years, we investigate the associations of threat and deprivation experiences with cortical thickness and surface area as well as amygdala and hippocampus volume. We hypothesize that threat will be associated with reduced thickness and surface area of cortical regions in the salience and limbic network and decreased volume of the amygdala and hippocampus. Additionally, we hypothesize that deprivation will be associated with reduced cortical thickness and surface area in the frontoparietal control network. Finally, we hypothesize that brain structure differences associated with threat will persist after controlling for deprivation and those associated with deprivation will persist after controlling for threat.

## 2 Method

### 2.1 Sample

Data were drawn from a larger study of youth examining maltreatment and emotion regulation in Seattle, WA between January 2015 and June 2017. A total of 161 youth participated in the MRI visit that serves as the basis of this paper. The MRI sample was comprised of children who were exposed to maltreatment, and a control sample of participants matched to each maltreatment-exposed participant on age, sex, and handedness. Exclusion criteria included IQ< 80, pervasive developmental disorder, active psychotic symptoms, mania, substance abuse, MRI contraindications (e.g., braces), or safety concerns as measured reported by caregivers during screening or assessed during the study visits. Brain structure data from 12 participants was excluded after data collection due to motion-related artifacts in their structural scan (see MRI pre-processing). Recruitment was targeted at identifying children with maltreatment experiences (see supplement for further recruitment details). See Table 1, Table 2 for socio-demographic characteristics of the sample with comparison to the Seattle population.

**Table 1:** Sample Description.

	mean	sd.	min	max
Age	12.64	2.67	8.03	17.25
Income-to-Needs Ratio	3.73	2.78	0.10	10.35
Threat Count	1.39	1.41	0	4.00
Deprivation Count	0.92	1.05	0	4.00
VEX-R Violence Experiences	3.58	2.66	0	10.00

**Table 2:** Sample Categorical Descriptors. a: Census data from the National Center on Educational Statistics (2015) – see supplement for detail. b: Many participants identified with more than one race/ethnicity descriptor.

		Sample (n = 149)		With Exclusions (n = 161)		Census Comparison^a^
Sex	Female	72	48.3%	77	47.8%	48.7%
	Male	77	51.7%	84	52.2%	51.3%
Parent Ed.	High School or Less	19	12.8%	26	16.1%	11.1%
(highest)	Some College (no degree)	18	12.1%	22	13.7%	10.4%
	College Degree	34	22.8%	34	21.1%	41.7%
	Post-Graduate Degree	52	34.9%	52	32.3%	37.0%
	Did not report	26	17.4%	27	16.8%	N/A
Race/Ethnicity^b^	White	116	77.9%	123	76.4%	73.3%
	Black	33	22.1%	39	24.2%	16.5%
	Native American	13	8.7%	16	9.9%	2.6%
	Asian	19	12.8%	21	13.0%	20.1%
	Pacific Islander	0	0.0%	1	0.6%	1.2%
	Latinx	19	12.8%	20	12.4%	9.4%
	Biracial	9	6.0%	9	5.6%	16.0%
	Other	14	9.4%	14	8.7%	4.2%

The Institutional Review Board at the University of Washington approved all procedures. Legal guardians provided written informed consent; children provided written assent.

### 2.2 Measures

#### 2.2.1 Threat experiences

A multi-informant, multi-method approach was used for assessing children’s experiences of threat. A composite threat score used in prior work (e.g., Sumner et al., 2019) was computed based on children’s experiences of physical abuse, sexual abuse, domestic violence, emotional abuse, and other forms of interpersonal violence. Caregivers and youth reported on physical abuse, sexual abuse, and domestic violence on the UCLA PTSD Reaction Index (PTSD-RI; Steinberg et al., 2013). Youth reported on experiences of physical abuse, sexual abuse, witnessing domestic violence, and emotional abuse on the Childhood Experiences of Care and Abuse Interview (CECA; Bifulco et al., 1994). Experiences of physical, sexual, and emotional abuse were also considered present if children scored above a validated threshold on the respective subscales from Childhood Trauma Questionnaire (CTQ; Bernstein et al., 1997; Walker, Gelfand et al., 1999). Domestic violence was also considered present if youth endorsed witnessing violence directed at a caregiver on the Violence Exposure Scale for Children Revised (VEX-R; Fox and Leavitt, 1995) or PTSD-RI. The number of different types of other witnessed or experienced interpersonal violence experiences (e.g., experiences of violence in the school or community) were measured based on youth report on the VEX-R. Finally, caregivers reported on their child’s experiences of physical abuse, sexual abuse, and domestic violence on the Juvenile Victimization Questionnaire (JVQ; Finkelhor et al., 2005). The final threat composite was computed by summing dichotomous scores for exposure to physical abuse, sexual abuse, domestic violence, and emotional abuse with the standardized interpersonal violence score from the VEX-R (See Supplement for example items and scoring). Inter-rater reliability was good for child and caregiver maltreatment reports (82.0% agreement; kappa=0.62).

#### 2.2.2 Deprivation experiences

A composite score reflecting youths’ experiences of physical or emotional neglect, food insecurity, or low levels of cognitive stimulation in the home was computed based on a similar multi-reporter, multi-method approach utilizing youth and caregiver report on several self-report and interview measures (Bifulco et al., 1994, Blumberg et al., 1999, Mott, 2004, Walker et al., 1999). Physical neglect was assessed based on youth report on the physical neglect subscale from the CTQ. Experiences of emotional neglect were scored as present based on youth-report on the emotional needs subscale of the CECA. Food insecurity was assessed using caregiver report on four questions from the short form of the U.S. Department of Agriculture’s Food Security Scale, a validated measurement of food insecurity (Blumberg et al., 1999). These items have been previously used in epidemiological surveys of youth psychopathology (e.g. Kessler et al., 2009). Cognitive stimulation was assessed using the short form of the Home Observation for Measurement of the Environment (HOME-SF; Mott, 2004). The HOME-SF has slightly different versions for children aged 6–9 and 10–15 years, with 16 items that are identical across these age ranges. We used only the 16 questions that are present in the HOME-SF for both younger and older children. The measure was scored using the cut-offs used in the original HOME, and following prior work (e.g., Sumner et al., 2019). Participants scoring under a 12 were considered to have been exposed to low levels of cognitive stimulation. The final deprivation composite was computed by summing the dichotomous scores for physical neglect, emotional neglect, food insecurity, and cognitive deprivation (See Supplement for example items and scoring).

#### 2.2.3 Socioeconomic Status (SES)

Household income was assessed by parent report using questions adapted from the U.S. Census Bureau Current Population Survey (U.S. U.S. Census Bureau, 2015). The income-to-needs ratio was calculated by dividing this approximate continuous income by the 2018 federal poverty line for a family of the reported size, such that a value less than one indicating that a family was living below the poverty line (e.g., $25,465 for a two parent, two child household). This value was then log transformed and used as the index of family SES, which was included as a control variable in all models.

### 2.3 Image acquisition and pre-processing

Scanning was performed on a 3 T Phillips Achieva scanner at the University of Washington Integrated Brain Imaging Center using a 32-channel head coil. T1-weighted MPRAGE volumes were acquired (repetition time = 2530 ms, TE=3.5 ms, flip angle=7°, FOV=256 ×256, 176 slices, in-plane voxel size=1 mm^3^).

Standard procedures, including cortical surface reconstruction, cortical and subcortical segmentation, and estimation of cortical thickness and surface area, were conducted using the FreeSurfer image analysis suite (Version 5.3; http://surfer.nmr.mgh.harvard.edu). The boundaries between grey and white matter and between grey matter and the pial surface were carefully inspected for each subject by at least two investigators and edited to ensure accuracy.

### 2.4 Whole brain analysis

In order to examine whether the associations between childhood adversity and neurodevelopmental outcomes were general or specific to particular dimensions of adverse experience, we estimated three linear models, first examining the association of threat and deprivation experiences with cortical thickness and surface area separately, and then examining the effect of each dimension while controlling for the other in order to examine their potential unique contributions to cortical thickness and surface area. This approach has been recommended (McLaughlin et al., 2021) and implemented in previous research (Sumner et al., 2019). All models included participants’ sex, age, and income-to-needs ratio as covariates. A final model was constructed including the interaction of threat and deprivation, respectively, with age. Cluster-wise correction was performed using a permutation approach implementing the Ter Braak approximation to correct for design non-orthogonality, as parametric approaches to cluster-wise correction have been shown to produce inflated false-positive rates (Greve and Fischl, 2018). Cluster forming and family-wise error rate were set at.05 (see Supplement for analysis code).

### 2.5 Subcortical analysis

Amygdala and hippocampus volume were extracted from the subcortical segmentation in Freesurfer (Fischl et al., 2002). Given that there is no consistent pattern of lateralization in findings of the relation between childhood violence exposure and hippocampus or amygdala volume (Hanson et al., 2015, McLaughlin et al., 2016, McLaughlin et al., 2019), right and left volumes were summed to create bilateral volumes. Sensitivity analyses examining left and right subcortical volumes separately are presented in the supplement. Similar to the whole brain analysis, each subcortical region of interest was first separately regressed against the threat and deprivation composite scores, controlling for age, sex, total intra-cranial volume, and income-to-needs ratio. Then, threat and deprivation were entered as regressors together with the same covariates to examine any potential differential associations with subcortical volume. A final set of models additionally included the interaction of threat and deprivation, respectively, with age. Linear modeling was performed in the r package ‘lavaan’ and missing data from MRI exclusion was accounted for using full-information maximum-likelihood estimation (FIML; Rosseel, 2012).

## 3 Results

**Fig. 1:**
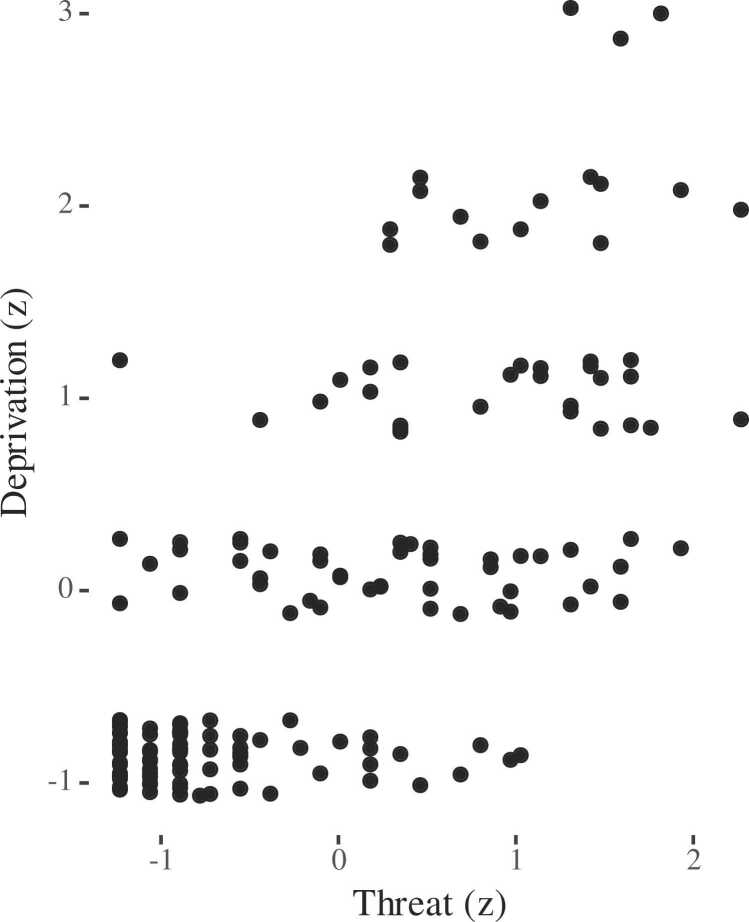
Distribution of threat and deprivation experience composite scores (r = 0.7). Deprivation scores have been jittered for legibility.

Relative distribution of threat and deprivation composite scores are shown in Fig. 1. Bivariate correlations between study variables are shown in Table 3.

**Table 3:** Bivariate Correlations. * : < 0.05; **: < 0.01; *** : < 0.001.

	1	2	3	4	5	6
1. Age						
2. Sex	0.14					
3. Income-to-Needs (log)	0.02	0.12				
4. Threat Composite	0.09	0.04	-0.54 ***			
5. Deprivation Composite	0.09	0.00	-0.50 ***	0.70 ***		
6. Hippocampal Volume	0.03	-0.35 ***	0.18 *	-0.22 **	-0.13	
7. Amygdala Volume	0.10	-0.38 ***	0.09	-0.24 **	-0.12	0.76 ***

### 3.1 Dimensions of adversity and cortical structure

#### 3.1.1 Cortical thickness

**Fig. 2:**
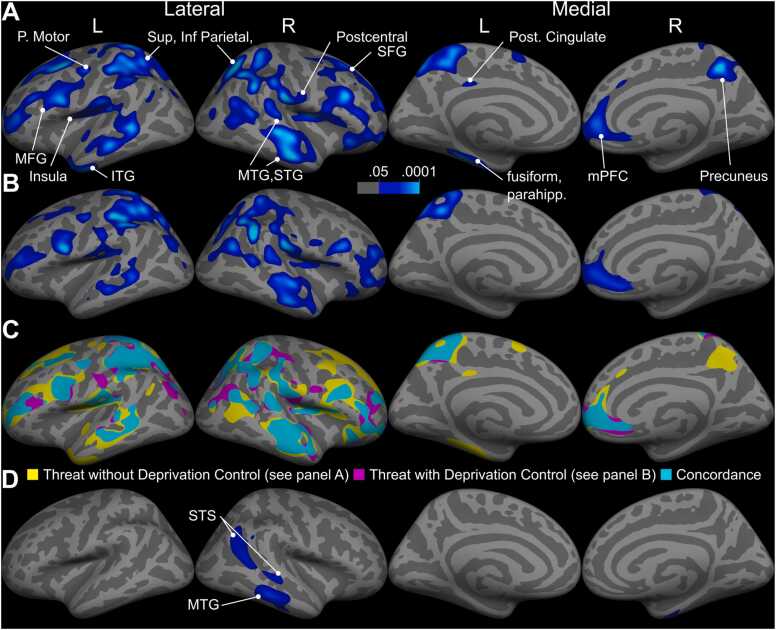
A: Map of p-values where cortex thickness was negatively associated with threat experiences, without control for deprivation. B: Map of p-values where cortex thickness was negatively associated with threat experiences, after controlling for deprivation. C: Concordance between A and B. D: map of p-values where cortical surface area was negatively associated with threat experiences after controlling for deprivation.

Greater experiences of threat were associated with thinner cortex in numerous regions, after controlling for sex, age, and income-to-needs ratio (see Fig. 2). Higher threat scores were associated with thinner cortex in regions comprising the salience (bilateral insula), limbic (right vmPFC), somatomotor (bilateral post-central gyrus, parietal operculum, and left precentral gyrus), default (bilateral middle and superior temporal cortex, left parahippocampal cortex, and right inferior frontal gyrus), visual (fusiform gyrus), and frontoparietal (bilateral superior frontal gyrus [SFG], middle frontal gyrus [MFG], superior and inferior parietal cortex, precuneus, and left posterior cingulate) networks.

Deprivation was not associated with differences in cortical thickness after controlling for sex, age, and income-to-needs ratio. In a sensitivity test, this null finding remained when deprivation was modeled without control for the income-to-needs ratio.

In a combined model, greater threat experiences continued to be associated with reduced thickness across numerous cortical regions after controlling for co-occurring deprivation experiences (see Fig. 2). Compared to the threat-only model, thinning associated with greater threat experiences (controlling for deprivation) was attenuated in the frontoparietal network, with reduced extent in bilateral MFG and left SFG and no associations with right SFG, bilateral precuneus, left posterior cingulate, as well as the default network, with reduced extent in superior temporal cortex and no association with left inferior temporal gyrus. Associations between threat and cortical thinning were otherwise similar with and without control for deprivation experiences.

#### 3.1.2 Cortical surface area

Threat experiences were associated with reduced surface area in portions of the right middle temporal gyrus and superior temporal sulcus after controlling for deprivation (see Fig. 2). No other associations of cortical surface area with threat or deprivation experiences were found in any model.

#### 3.1.3 Adversity and age-related change in cortical structure

No associations were observed between age and either threat or deprivation in relation to cortical thickness or surface area. We additionally found no evidence for developmental differences in the associations of threat or deprivation experiences with cortical thickness or surface area when using pubertal stage as the metric of development instead of age (see supplement for details).

### 3.2 Dimensions of adversity and sub-cortical volume

Greater threat experiences were associated with reduced volume of the amygdala (*ß* = −0.18, *p* = .021, 95% CI [−0.34, −0.03]), but not hippocampus (*ß* = −0.05, *p* = .481, 95% CI [−0.21,0.10]), after controlling for age, sex, income-to-needs ratio, and intra-cranial volume. Deprivation experiences were not associated with either amygdala (*ß* = −0.06, *p* = .441, 95% CI [−0.22,0.10]) or hippocampal (*ß* =0.01, *p* = .935, 95% CI [−0.14,0.15]) volume (see supplement Fig. 2).

In a model where both composite scores were entered, threat experiences continued to be associated with smaller amygdala volume (*ß* = −0.23, *p* = .021, 95% CI [−0.42, −0.03]), but not hippocampal volume (*ß* = −0.08, p = .390, 95% CI [−0.27,0.10]. Deprivation was not associated with either amygdala (*ß* =0.07, *p* = .459, 95% CI [−0.12,0.26]) or hippocampal (*ß* =0.05, *p* = .579, 95% CI [−0.13,0.23]) volume (see Table 4).

**Table 4:** Fully Specified Sub-Cortical Models; * : < 0.05; **: < 0.01; *** : < 0.001.

Model	Model Term	β	se	CI (lower)	(upper)	p
Hippocampal Volume (mm3)	Intercept	3.90***	0.94	2.01	5.71	< 0.001
	Deprivation	0.05	0.09	-0.13	0.23	0.579
	Threat	-0.08	0.10	-0.27	0.10	0.390
	Age	-0.07	0.07	-0.21	0.06	0.287
	Female	-0.05	0.08	-0.21	0.11	0.563
	Intra-cranial volume	0.57***	0.08	0.42	0.72	< 0.001
	Income-to-Needs	0.11	0.08	-0.05	0.28	0.181
Amygdala Volume (mm3)	Intercept	2.97**	0.98	1.05	4.88	0.002
	Deprivation	0.06	0.10	-0.13	0.25	0.551
	Threat × Age	0.18*	0.08	0.03	0.34	0.022
	Threat	-0.24*	0.10	-0.43	-0.05	0.014
	Age	-0.05	0.08	-0.21	0.12	0.578
	Female	-0.15	0.08	-0.32	0.01	0.065
	Intra-cranial volume	0.42***	0.08	0.26	0.58	< 0.001
	Income-to-Needs	-0.04	0.09	-0.21	0.13	0.637

#### 3.2.1 Adversity and age-related changes in subcortical volume

**Fig. 3:**
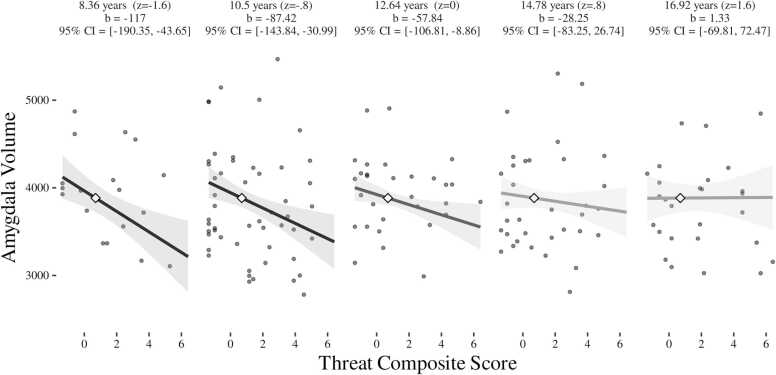
Age and Threat interact to predict Amygdala Volume (visualized using InterActive; McCabe et al., 2018).

Age interacted with threat to predict amygdala volume, such that younger participants with higher levels of threat showed smaller amygdala volume, but older participants did not show threat-related differences in amygdala volume (*ß* =0.18, *p* = .022, 95% CI [0.03,0.34]; see Fig. 3, Table 4). Fit indices of this interaction model were superior to the model without an age interaction (χ^2^ (1) = 5.11, *p* = .024; see supplement for detail). The interaction of age with threat on hippocampal volume was marginal (*ß* =0.15, *p* = .056, 95% CI [0,0.29]), and the pattern was in the same direction as amygdala with threat associated with smaller hippocampal volume among children but not adolescents (see Supplement Fig. 3).

## 4 Discussion

The present study examined associations between experiences of childhood adversity involving threat and deprivation—independently and jointly—with cortical thickness and subcortical volume. Greater experiences of early-life threat were associated with numerous structural differences, including in brain regions typically recruited during salience processing, perception, and self-reflection. Structural differences in salience processing areas included thinning in bilateral anterior insula and right vmPFC, as well as reduced volume in the amygdala among younger, but not older, participants. Cortical regions involved in perception that were associated with threat-related experiences included the ventral visual stream (right fusiform and inferior temporal cortex), primary and secondary somatosensory cortex (left post-central gyrus and bilateral parietal operculum), and auditory processing areas (bilateral superior temporal cortex). Several regions of the default network were thinner and/or had reduced surface area in children with greater threat-related experiences, including medial PFC and lateral temporal cortex. We additionally observed thinning associated with threat in the frontoparietal network including bilateral middle frontal gyrus, precuneus, superior frontal gyrus, left parietal cortex, and inferior pre-central gyrus. After controlling for deprivation, similar associations between threat and cortical thickness were observed, but with reduced extent primarily in frontoparietal regions, including middle and superior frontal gyrus, as well as reductions in associations of threat with cortical surface area in the right middle temporal cortex and superior temporal sulcus. We did not find an association of deprivation experiences with brain structure with or without control for threat.

The associations we observed between threat experiences and structural differences in the salience processing and limbic networks were consistent with our hypotheses and previous literature. These networks signals the affective salience of stimuli to the rest of the brain in order to organize rapid responses to the environment (Fusar-Poli, 2009, Öhman, 2005). Structural changes in these networks have been extensively documented in children with threat-related experiences (Gold et al., 2016, Kelly et al., 2013, Lim et al., 2014, McLaughlin et al., 2016, McLaughlin et al., 2019). Behavioral studies have demonstrated that youth who have experienced violence prioritize threat-related information during information processing (Pollak and Sinha, 2002, Pollak and Tolley-Schell, 2003), suggesting heightened sensitivity of these brain networks to environmental cues that could signify the presence of threat. Future research should explore whether the structural changes we observed mediate the elevated neural and behavioral responses to threat commonly observed in children who have experienced violence (see McLaughlin et al., 2020; McLaughlin and Lambert, 2017 for reviews). Given that cortical thinning in these networks is typically observed during adolescence, the thinning we observed in anterior insula and medial PFC may represent accelerated development of key regions involved in salience and emotional processing (Callaghan and Tottenham, 2016, Colich et al., 2020). Alternatively, thinning in these networks may simply reflect circuit refinement (i.e. synaptic pruning and/or increased myelination) related to more frequent utilization of these regions (Tau and Peterson, 2010). Further research will be needed to investigate the development of these differences in longitudinal samples and associations with relevant emotional and behavioral processes.

We also observed smaller amygdala volume associated with greater experiences of threat, although this was restricted to the children in our sample and not the adolescents. Smaller amygdala volume in children who have experienced violence is consistent with prior literature (see McLaughlin et al., 2019; Teicher and Samson, 2013 for review), and complements our findings of threat-related thinning in the related salience and limbic networks. However, the age-related differences we observed in the association of threat with amygdala volume differ from prior work in longitudinal samples. In typically developing samples, amygdala volume increases throughout childhood until late adolescence/early adulthood (Russell et al., 2021, Wierenga et al., 2014). While the association of adversity with amygdala development over time is a subject of ongoing investigation, Whittle et al. (2013) found that during the transition from early to mid-adolescence, youth with more severe child maltreatment experiences showed equivalent or increased baseline amygdala volumes but slower growth in amygdala volume relative to youth with less severe maltreatment experiences. In our sample, most participants experienced violence relatively early in development (prior to the age of 8 years), which might have contributed to the differences in amygdala volume we observed in childhood here. It is unclear why these differences were not present in the adolescents in our sample. Importantly, the present study was cross-sectional, which limits its ability to investigate change in brain structure across age. Further longitudinal work is needed to explore how threat experiences relate to structural changes in the amygdala across development.

Youth with more experiences of threat also showed thinner cortex in a broad network of regions associated with perception across multiple sensory modalities. These findings align with previous studies reporting structural changes in areas of the brain implicated in perception among individuals with a history of threat-related adversity. For example, reductions in thickness of visual processing areas have been reported in young adults who witnessed domestic violence as children (Tomoda et al., 2012), and reductions in somatosensory cortex thickness have been observed among adults with previous exposure to sexual abuse (Heim et al., 2013) as well as maltreated children (Kelly et al., 2015). Functionally, perceptual networks play an important role in salience processing. In a meta-analysis of neural responses to affective stimuli, Satpute et al. (2015) demonstrated that affect inductions utilizing unpleasant stimuli are typically accompanied by enhanced neural activity in salience processing regions (e.g. amygdala, hippocampus, and anterior insula) as well as primary and secondary sensory processing areas, as compared to neutral stimuli, according to the sensory modality in which the affective stimuli were administered (e.g. ventral visual stream for unpleasant images, superior temporal cortex for unpleasant sounds). Given the role of perception networks in salience processing, the thinning we observed in these areas could reflect similar developmental processes as that observed in the core salience network. However, such a conclusion is premature, and further research will be needed to discern the causes and functional consequences of structural differences in these areas associated with threat. Future research should additionally explore whether thinning in somatosensory areas contributes to the reduced emotional awareness often observed in youth who have experienced violence (Weissman et al., 2019, Weissman et al., 2020), as somatosensory cortex has been shown to play a role in interoception and emotion awareness (Damasio et al., 2000, Kropf et al., 2018, Liddell et al., 2005, Straube and Miltner, 2011).

Youth with greater numbers of threat experiences also showed thinning and reduced surface area in the default network, including medial PFC and lateral temporal cortex. These regions are frequently recruited during tasks involving self-reflection and social cognition (e.g., Dixon et al., 2017; Hein and Knight, 2008). Threat-related thinning in these regions parallels recent findings of functional differences in these regions that are associated with experiences of threat, but not deprivation (Weissman et al., 2022). Future work should explore whether these differences mediate previously observed social information processing differences in children who have experienced violence (McLaughlin et al., 2020), such as reduced theory of mind performance (Deen et al., 2015, Heleniak and McLaughlin, 2020).

Finally, threat was associated with cortical thinning and reduced surface area in the frontoparietal control network (Yeo et al., 2011). The thinning we observed in this network contradicted our hypothesis that thickness in these areas would be uniquely associated with deprivation. Importantly, association between threat experiences and thinning in PFC was reduced substantially when controlling for deprivation experiences, suggesting that at least some of the thinning observed was not specific to threat-related experiences.

Outside the frontoparietal network, the structural differences we observed in threat-exposed youth remained after controlling for co-occurring deprivation, suggesting that our results reflected distinct developmental processes related to threat-related experiences rather than stress or childhood adversity in general. This interpretation is consistent with prior work showing that threat experiences may have effects on salience processing that are distinct from other forms of adversity (Lambert et al., 2017, Machlin et al., 2019, Sheridan et al., 2019, Weissman et al., 2022).

We did not find evidence of associations between brain structure and deprivation. Associations between deprivation and cortical structure have been observed in samples exposed to severe deprivation experiences including institutional rearing (Bick and Nelson, 2016, Herzberg et al., 2018, Hodel et al., 2015) and neglect (Edmiston et al., 2011). Although we measured neglect experiences, it may be that the deprivation experiences among children in our sample were not severe enough to have led to similar cortical changes. However, prior work on cognitive stimulation has observed thinning in the frontoparietal network in children who experienced reductions in cognitive stimulation in the normative range (Rosen et al., 2018).

Although this study had many strengths, limitations of the study design and sample should be considered in interpreting these results. The study was cross-sectional by design and these correlational findings cannot be used to make inferences about causation or developmental differences over time. Our interpretations as to the function of observed structural changes are speculative and will need to be tested in future work. We did not find evidence for any of our hypotheses on the association of brain structure with deprivation. Additionally, we predicted that deprivation would be specifically associated with thinning in the frontoparietal control network but instead found an association between threat and thinning in these regions. Moreover, we have previously proposed that cognitive deprivation may drive the reduced cortical thickness and surface area in the ventral visual stream that has been observed among low-SES children (Noble et al., 2015, Rosen et al., 2018), but instead found an association between these regions and experiences of threat. Importantly, our recruitment strategies specifically targeted threat-exposed youth. Consequently, threat and deprivation were significantly co-occurring in our sample, and threat-related experiences were more common than deprivation-related experiences. Additionally, we had fewer measures of deprivation than threat. Our deprivation composite score was sensitive to several indices of deprivation (i.e., physical and cognitive deprivation) that may have different associations with brain structure. These limitations reduced our power to detect deprivation-related associations. Furthermore, recent work has suggested that very large samples are required to reliably identify associations between cortical thickness and phenotypic variables with adequate control for false positives (Marek et al., 2022). Although this is among the largest samples examining associations of trauma with neural structure in children to date, it is small by the standards identified in Marek and colleagues (2022). Small sample sizes have undoubtedly contributed to heterogeneity in reported associations between dimensions of adversity and brain structure (McLaughlin et al., 2019). Future studies including large samples and a wide range of exposure histories should be conducted to further explore the associations between experiences of threat and deprivation with brain structure. Because of the difficulty of recruiting such a sample, data-pooling efforts across institutions are underway to facilitate these analyses (McLaughlin et al., 2019).

## 5 Conclusions

We observed that exposure to threatening experiences, but not experiences of deprivation, was associated with thinner cortex in youth exposed to threat in numerous cortical regions involved in salience processing, self-reflection, and perceptual processing, as well as smaller subcortical volume in the amygdala (among younger participants). These results provide further evidence that childhood trauma has pervasive influences on structural brain development. Structural differences in salience processing areas may contribute to changes in behavior in children exposed to threatening experiences, such as enhanced sensitivity and reactivity to threatening stimuli. Further research should evaluate this possibility, as enhanced sensitivity to threat may contribute to the increased risk for psychopathology experienced by children who have experienced violence.

## Declaration of Competing Interest

The authors declare that they have no known competing financial interests or personal relationships that could have appeared to influence the work reported in this paper.

## Data Availability

Data will be made available on request.
