# Supplementary material for: Childhood trauma and brain structure in children and adolescents
Source: Dev Cogn Neurosci. 2022 Nov 23;59:101180. doi: 10.1016/j.dcn.2022.101180 (PMC9800267; doi:10.1016/j.dcn.2022.101180)
Supplement: Supplementary file 1 — Supplementary material. [file mmc1.pdf]

Childhood Trauma and Brain Structure in Children and Adolescents: Supplemental methods

Matthew Peverill, Maya L. Rosen, Lucy A. Lurie, Kelly A. Sambrook, Margaret A Sheridan, and Katie A. McLaughlin

Abstract

This document provides supplemental text to the main manuscript. It presents methadological details, our statistical code, formulas, and background information necessary for transparency and replication, including supplemental analyses and figures.

Contents

1 Supplemental Methods **2**

1.1 Recruitment . . . 2

1.2 Assessment of Deprivation and Threat . . . 2

2 Analysis Code **8**

2.1 Sample Description (Tables 1 and 2) . . . 8

2.2 Table 3: Bivariate Correlation Table . . . 9

2.3 Whole brain analyses . . . 9

2.4 Subcortical Models . . . 12

2.5 Figure generation code . . . 14

3 Supplemental Analyses **15**

3.1 Sub-cortical Laterality Tests . . . 15

3.2 Pubertal Timing Interaction Analysis . . . 19

3.3 Main effect of exposure on subcortical volumes. . . . 21

3.4 Age × Threat Interaction on Hippocampal Volume . . . 23

4 R Environment **25**

5 Supplemental References **26**

1 Supplemental Methods

1.1 Recruitment

Participants were youth (8-17) living in the Seattle area who were recruited for a study examining neural development in youths with and without exposure to violence. Youth and caregivers were recruited for participation at schools, after-school and prevention programs, adoption programs, food banks, shelters, parenting programs, medical clinics, and the general community between January 2015 and June 2017.

Recruitment efforts were targeted at recruiting a sample with ample variability in exposure to violence. To that end, we recruited from neighborhoods with high levels of violent crime, clinics that served predominantly low-SES catchment areas, and agencies that worked with families who have been victims of violence (e.g., domestic violence shelters, programs for parents mandated to receive intervention by Child Protective Services). Inclusion criteria for the violence-exposed group included exposure to physical or sexual abuse or direct witnessing of domestic violence. Children in the control group were matched to children in the violence-exposed group on age, sex, and handedness; inclusion criteria required an absence of exposure to significant interpersonal violence. Exclusion criteria included IQ < 80, presence of pervasive developmental disorder, active psychotic symptoms or mania, active substance abuse, and presence of safety concerns. Participants who completed the MRI visit also met standard MRI inclusion criteria (i.e., absence of braces, claustrophobia).

1.2 Assessment of Deprivation and Threat

Example questions for deprivation and threat measures are listed in tables S1 and S2.

Table S1: Threat Exposure Measures

Threat Experience	Measures
Physical Abuse	**CECA:** E.g. "When you were a child or teenager were you ever hit repeatedly with something or punched, kicked, or burnt by someone in the household?" (Absent = no; Present = yes)<br>**CTQ:** Physical Abuse Subscale: Absent = 7; Present = 8+ E.g.: "People in my family hit me so hard that it left me with bruises or marks"<br>**JVQ:** E.g. "Not including spanking on your child's bottom, at any time in your child's life, did a grown-up in your child's life hit, beat, kick, or physically hurt your child in any way?" (Absent = no; Present = yes)<br>**PTSD-RI:** E.g. "Being hit, punched, or kicked very hard at home (do not include ordinary fights between siblings)" (Absent = no; Present = yes)
Sexual Abuse	**CECA:** E.g. "Has someone ever touched your private parts when you didn't want them to?" (Absent = no; Present = yes)<br>**CTQ:** E.g. Sexual Abuse Subscale: Absent = 5; present = 6+ E.g.: "Someone tried to touch me in a sexual way or tried to make me touch them."<br>**JVQ:** Sexual Victimization Subscale: Absent = 0; Present = 1+<br>**PTSD-RI:** E.g. "Having an adult or someone much older touch their private, sexual body parts." (Absent = no; Present = yes)
Emotional Abuse	**CECA:** Emotional Abuse subscale: Absent 24; Present = 25+ Eg.: "My parent often picked on me unfairly."<br>**CTQ:** Emotional Abuse Subscale: Absent = 8; present = 9+ E.g. "People in my family said hurtful or insulting things to me."<br>**JVQ:** E.g. "At any time in your child's life, did your child get scared or feel really bad because grown-ups in your child's life called your child names, said mean things to your child, or said they didn't want your child?" (Absent = no; Present = yes)
Domestic Violence	**CECA:** E.g. "When you were a child or teenager, did you ever see or hear your parents or caregivers hit each other repeatedly with something (like a belt or stick) or hit, punch, kick, or burn each other?" (Absent = no; Present = yes)<br>**VEXR:** Absent = no exposure; Present = youth reported ever witnessing an object being thrown at a caregiver, a caregiver being pushed hard, a caregiver being chased, a caregiver being slapped hard, a caregiver being beat up, or a caregiver being threatened or injured with a weapon.<br>**JVQ:** E.g. "At any time in your child's life, did your child see a parent get pushed, slapped, hit, punched, or beat up by another parent, or their boyfriend or girlfriend?" (Absent = no; Present = yes)<br>**PTSD-RI:** E.g. "Seeing a family member being hit, punched, or kicked very hard at home (do not include ordinary fights between siblings)." (Absent = no; Present = yes)
Other Interpersonal Violence	**VEXR:** Absent = no exposure; Present = youth reported experiencing violent victimization from peers, family members, or other adults that was not otherwise defined as physical or sexual abuse or youth reported witnessing violence between peers, family members, or other adults that was not otherwise defined as witnessing domestic violence.

Table S2: Deprivation Exposure Measures

Deprivation Experience	Measures
Physical Neglect	**CTQ:** Physical Neglect Subscale: Absent = 7; Present = 8+ E.g. "I had to wear dirty clothes."
Emotional Neglect	**CECA:** Neglect Subscale: Absent 22; Present = 23+ E.g. "My parent tried to make me feel better when I was upset"
Cognitive Deprivation	**HOME-SF:** Cognitive Deprivation Subscale: Absent = 12+; Present = <12 E.g. "Did you and/or your partner teach your child numbers at home?"
Food Insecurity	**USDA Food Insecurity scale:** Absent 3; Present = 4+<br>In the past 12 months, were you ever hungry but did not eat because you could not afford to buy food?" (0 = no, 1 = yes)<br>"In the past 12 months, did you ever eat less than you felt you should because you didn't have money to buy food?" (0 = no, 1 = yes)<br>"How often in the past 12 months did you not have enough money to buy food?" (0 = never, 1 = rarely, 2 = sometimes, 3 = often)<br>"How often in the past 12 months could you not afford to eat balanced meals?" (0 = never, 1 = rarely, 2 = sometimes, 3 = often)

1.2.1 Exposure Scores – Calculation and Comparison to Component Measures

To calculate the threat composite score, the presence/absence of physical abuse, sexual abuse, emotional abuse, and exposure to domestic violence were were calculated across measures using an 'AND' rule and then summed to produce a count score. The interpersonal violence score from the VEX-R, which measures milder forms of threat exposure with greater variability, was then z-scored and added to the count score.

To calculate a deprivation composite score, dichotomous scores for physical neglect, emotional neglect, food insecurity, and low-cognitive stimulation were calculated across measures using an 'AND' rule and then summed.

Use of multi-modal interviews and self-report measures conducted simultaneously with children and caregivers is the gold standard approach to maltreatment and adversity assessment (Widom, Raphael, DuMont, 2004). Such an approach is important because children and caregiver reports of violence exposure frequently do not align. Under-reporting of these experiences is the primary driver of informant discrepancies (see Hardt & Rutter, 2004; Baldwin et al., 2019 for reviews on recall bias related to maltreatment reporting). The composite scores we created demonstrated superior sensitivity relative to their component measures (see Figures S1, S2)

```
df$CTQ_COUNT <-
    rowSums(df[, c("CTQ_PHYSICAL", "CTQ_SEXUAL", "CTQ_EMOTIONAL")])
df$CECA_COUNT <-
    rowSums(df[, c("CECA_PHYSICAL", "CECA_SEXUAL", "CECA_EMOTIONAL", "CECA_DV")])
df$JVQ_COUNT <-
    rowSums(df[, c("JVQ_PHYSICAL", "JVQ_SEXUAL", "JVQ_EMOTIONAL")])
df$UCLACHILD_COUNT <-
    rowSums(df[, c("UCLA_PHYSICAL", "UCLA_SEXUAL", "UCLA_DV")])
df$UCLAPARENT_COUNT <-
    rowSums(df[, c("UCLA_PARENT_PHYSICAL",
                               "UCLA_PARENT_SEXUAL",
                               "UCLA_PARENT_DV")])

threatcounts <- df %>%
```

```
    select(threat,CTQ_COUNT:UCLAPARENT_COUNT,VEXR_DV,VEX_NUM_VIOLENCE) %>%
    filter(threat >= 1) %>%
    arrange(threat) %>%
    rowid_to_column(var="threatorder") %>%
    mutate(VEX_NUM_VIOLENCE = pmax(scale(VEX_NUM_VIOLENCE),0)) %>%
    gather("meas","Count",-threatorder) %>%
    mutate(meas = recode(meas, threat = "Threat Composite",
                          CTQ_COUNT = "CTQ",
                          CECA_COUNT = "CECA",
                      JVQ_COUNT = "JVQ",
                          UCLACHILD_COUNT = "PTSD-RI Child",
                          UCLAPARENT_COUNT = "PTSD-RI Caregiver",
                          VEXR_DV = "VEX-R DV",
                          VEX_NUM_VIOLENCE = "VEX-R IPV"))

## Figure S1
ggplot(threatcounts,aes(x=threatorder,y=meas,fill=Count)) +
    geom_tile() +
    xlab("Threat composite score") +
    scale_fill_gradient(low="white", high="black") +
    theme(axis.title.y = element_blank(),
            axis.ticks.x = element_blank(),
            axis.text.x = element_blank(),
            legend.position = "bottom")
```

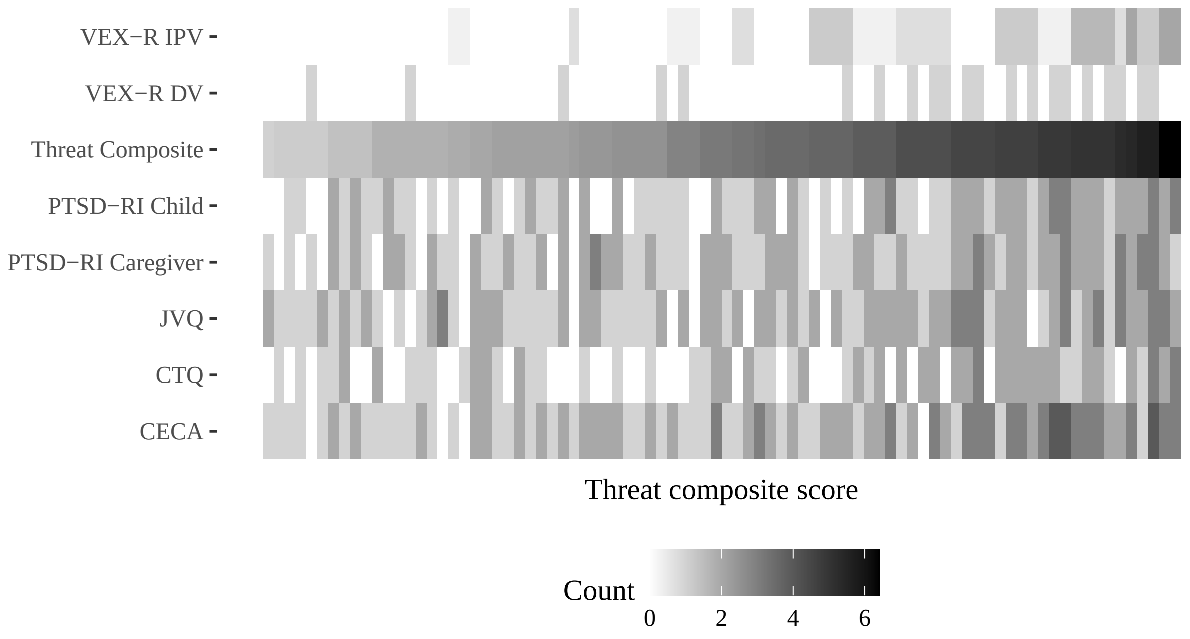

Figure S1: Comparison of threat composite to component measures. No one measure captures all variability.

```
df$HOME_rev<-df$HOME_TOT*-1
depcounts <- df %>%
    mutate(FOOD_INSECURE=as.numeric(FOOD_INSECURITY_CONT>0)) %>%
    select(NUM_DEP,PHYSICAL_NEGLECT,CECA_NEGLECT,COG_DEP,FOOD_INSECURE) %>%
    filter(NUM_DEP > 0) %>%
```

```
    arrange(NUM_DEP) %>%
    rowid_to_column(var="deporder") %>%
    gather("meas","Count",-deporder) %>%
    mutate(meas = recode(meas, NUM_DEP = "Deprivation Composite",
                         PHYSICAL_NEGLECT = "CTQ Physical Neglect",
                         CECA_NEGLECT = "CECA Neglect",
                     COG_DEP = "HOME-SF < 12",
                         FOOD_INSECURE = "Any Food Insecurity",
                         ))

ggplot(depcounts,aes(x=deporder,y=meas,fill=Count)) +
    geom_tile() +
    xlab("Deprivation composite score") +
    scale_fill_gradient(low="white", high="black") +
    theme(axis.title.y = element_blank(),
              axis.ticks.x = element_blank(),
              axis.text.x = element_blank(),
              legend.position = "bottom")
```

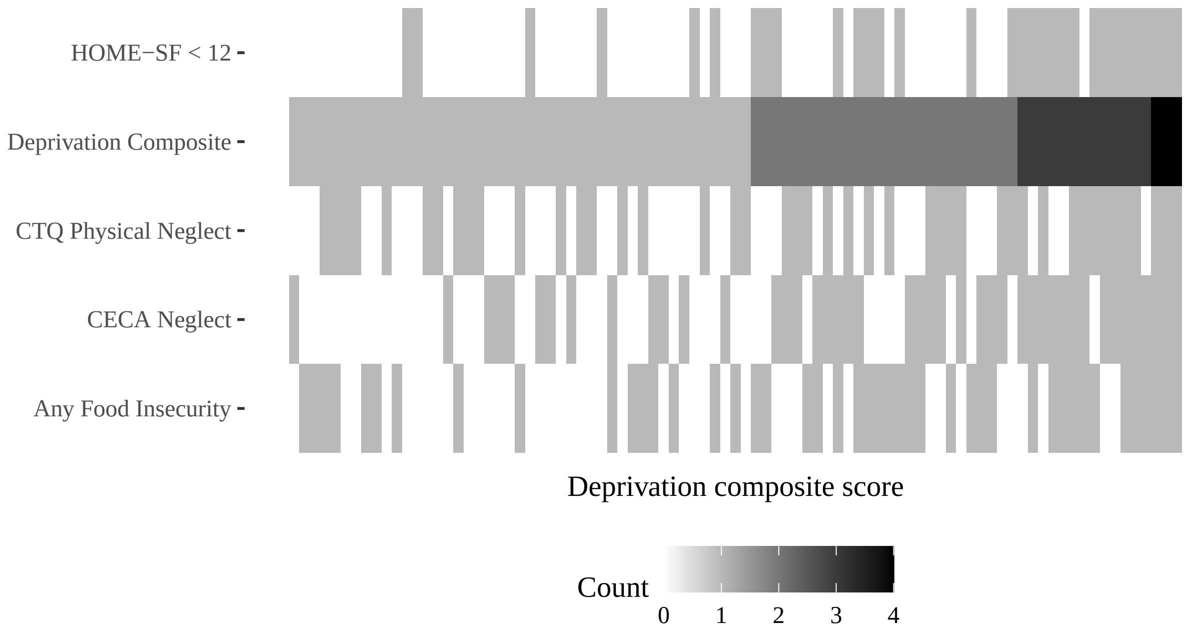

Figure S2: Comparison of deprivation composite to component measures. No one measure captures all variability.

1.2.2 Internal Validity of Composite Scores

The threat composite score showed strong correlations with its component measures. It was correlated to the endorsement of DV (only) on the VEX-R at $r = .47$ and all other component instruments at $r > .69$ (See table S3).

```
thrcortable<-sdcorr(
    df[, c(
        "threat",
```

```
        "CTQ_ABUSE",
        "CTQ_COUNT",
        "CECA_COUNT",
        "JVQ_COUNT",
        "UCLACHILD_COUNT",
        "UCLAPARENT_COUNT",
        "VEXR_DV",
        "VEX_NUM_VIOLENCE"
    )],
    labels = c(
        "Threat Composite",
        "CTQ Abuse Severity",
        "CTQ Abuse Count",
        "CECA Abuse/DV Count",
        "JVQ Abuse Count",
        "UCLA Abuse/DV Count -- Child",
        "UCLA Abuse/DV Count -- Parent",
        "VEXR DV Yes/No",
        "VEXR IPV Score"
    ),starAttach = TRUE
)
```

Table S3: Correlation of Threat Composite with Components

	1	2	3	4	5	6	7	8
1. Threat Composite								
2. CTQ Abuse Severity	.66***							
3. CTQ Abuse Count	.69***	.9***						
4. CECA Abuse/DV Count	.85***	.69***	.66***					
5. JVQ Abuse Count	.75***	.55***	.56***	.72***				
6. UCLA Abuse/DV Count – Child	.76***	.62***	.64***	.84***	.68***			
7. UCLA Abuse/DV Count – Parent	.79***	.54***	.56***	.79***	.74***	.75***		
8. VEXR DV Yes/No	.47***	.34***	.29***	.46***	.42***	.43***	.4***	
9. VEXR IPV Score	.89***	.53***	.53***	.66***	.54***	.58***	.54***	.42***

The deprivation composite score also showed strong correlations with its component measures, with all $r > .59$ (see table S4).

```
depcortable<-sdcorr(
    df[, c("dep",
             "CTQ_PHSYNEG",
             "CECA_NEGLECT",
             "HOME_rev",
             "FOOD_INSECURITY_CONT")],
    labels = c(
        "Deprivation",
        "CTQ Physical Neglect Severity",
        "CECA Neglect (dichotomous)",
        "HOME-SF Total Score",
        "USDA Food Insecurity Scale"
```

```
    ),starAttach = TRUE
)
```

Table S4: Correlation of Deprivation Composite with Components

	1	2	3	4
1. Deprivation				
2. CTQ Physical Neglect Severity	.59***			
3. CECA Neglect (dichotomous)	.7***	.35***		
4. HOME-SF Total Score	.67***	.34***	.32***	
5. USDA Food Insecurity Scale	.59***	.08	.21**	.43***

2 Analysis Code

2.1 Sample Description (Tables 1 and 2)

Table one provides descriptors of continuous variables:

```
library(tables)
# Functions for demographics table:
Mean <- function(x) base::mean(x, na.rm=TRUE)
Sd <- function(x) stats::sd(x, na.rm=TRUE)
Min <- function(x) base::min(x, na.rm=TRUE)
Max <- function(x) base::max(x, na.rm=TRUE)

tabular<-tabular(S3AGE + INC_NEEDS + NUM_ABUSE + NUM_DEP + VEX_NUM_VIOLENCE
                ~ (Mean+Sd+Min+Max),
                data=df)

# write.csv(tabular,
#                    file = "table-sampledescription.csv",
#                    row.names = TRUE,
#                    col.names = TRUE)
```

Table 2 provides count data on race/ethnicity, sex, and parent education. We will include counts with and without exclusions for MRI image quality.

```
df$FEMALE.factor<-factor(df$FEMALE,c(1,0),c("female","male"))
df$cortsample<-!is.na(df$lh_medialorbitofrontal_thickness)

yescount<-function(x) {
    sum(x=="Yes",na.rm="TRUE")
}

tabular2a <-
    tabular((FEMALE.factor + DEGREEP1P2) * (length) ~ df$cortsample + 1, data =
                        df)
tabular2b <-
    tabular(
        (I(WHITE) + I(BLACK) + I(NATIVE_AMERICAN) + I(ASIAN) + I(PACIFIC_ISLANDER) +
```

```
         I(LATINO) + I(MIDDLE_EASTERN) + I(BIRACIAL) + I(OTHER)) * yescount
        ~ df$cortsample + 1,
        data = df
    )

# write.csv(tabular2a,
#                   file = "table-samplefactordescriptionA.csv",
#                   row.names = TRUE,
#                   col.names = TRUE)
# write.csv(tabular2b,
#                   file = "table-samplefactordescriptionB.csv",
#                   row.names = TRUE,
#                   col.names = TRUE)
```

Sample percentages were added using Microsoft Excel. Census Comparison figures were drawn from tables provided by the National Center for Education Statistics from the 2015-2019 American Community Survey data. Parent education data was taken from table PDP02.5 (Educational Attainment) in the 'Seattle Public Schools' geography for the population 'Total Parents of Children.' Child demographics were taken tables generated in the 'Seattle Public Schools' geography for the population 'Total Children'. Data was draw from tables CDP05.1 (sex and age), CDP05.2 (race; specifically the 'race alone or in combination with one or more other races section'), and CDP05.3 (Hispanic or Latino and Race).

27 participants did not report parent education of either parent (26 after exclusions)

2.2 Table 3: Bivariate Correlation Table

```
corrtable <-
    sdcorr(df[, c("S3AGE",
                                "FEMALE",
                                "logINR",
                                "threat",
                                "dep",
                                "Hippocampus.orig",
                                "Amygdala.orig")])
#write.csv(corrtable,file="table-correlation.csv")
```

2.3 Whole brain analyses

For all analyses, subjlist.fsgd is used with the following format:

```
GroupDescriptorFile 1
Title   subjlist
Class   Class1  plus    blue
Variables
Input   1001_1  Class1
Input   1002_1  Class1
[...]
DefaultVariable
```

2.3.1 Single Dimension Model Setup

A matrix ('.X') file is assembled from the following columns:

- Sex Male
- Sex Female
- Age (centered)
- Income-to-needs ratio (log transformed, centered), 10 missing values inputed at 0 (mean)
- Threat or deprivation composite score (centered and scaled)

e.g.:

```
+1.00000 +0.00000 +3.11124 +1.18250 -0.51867
+0.00000 +1.00000 -0.20820 +0.89148 -1.01776
+1.00000 +0.00000 -2.06654 +0.89148 -1.01776
+0.00000 +1.00000 +3.05846 +0.89148 -1.18412
+0.00000 +1.00000 +2.74735 +0.01935 -1.18412
+0.00000 +1.00000 +0.46124 +0.16790 -0.85139
+0.00000 +1.00000 -0.27487 +0.89148 -1.18412
+0.00000 +1.00000 +1.84457 +0.89148 -1.18412
+0.00000 +1.00000 +4.03346 +1.12215 +0.20590
+0.00000 +1.00000 -1.20543 -1.24707 -0.12682
[...]
```

For the exposure contrast, a contrast is then designed to measure the effect of the exposure variable.

e.g.:

```
0 0 0 0 1
```

2.3.2 Full Dimensional Model Setup

As above, but both exposures are included in the '.X' file. Two contrasts are then defined for each exposure controlling for the other, ie:

```
0 0 0 0 1 0
```

and

```
0 0 0 0 0 1
```

2.3.3 Full Dimensional Model with Age Interaction Setup

A matrix file is constructed including everything in the dual exposure model, as well as columns for 'threat x age' and 'deprivation x age.'

eg:

```
+1.00000 +0.00000 +3.11124 +1.18250 -0.51867 -0.85198 -1.61371 -2.65072
+0.00000 +1.00000 -0.20820 +0.89148 -1.01776 -0.85198 +0.21190 +0.17739
+1.00000 +0.00000 -2.06654 +0.89148 -1.01776 -0.85198 +2.10323 +1.76065
+0.00000 +1.00000 +3.05846 +0.89148 -1.18412 +0.08144 -3.62159 +0.24908
```

```
+0.00000 +1.00000 +2.74735 +0.01935 -1.18412 -0.85198 -3.25319 -2.34070
+0.00000 +1.00000 +0.46124 +0.16790 -0.85139 -0.85198 -0.39270 -0.39297
+0.00000 +1.00000 -0.27487 +0.89148 -1.18412 -0.85198 +0.32548 +0.23418
+0.00000 +1.00000 +1.84457 +0.89148 -1.18412 -0.85198 -2.18420 -1.57155
+0.00000 +1.00000 +4.03346 +1.12215 +0.20590 -0.85198 +0.83050 -3.43644
+0.00000 +1.00000 -1.20543 -1.24707 -0.12682 +0.08144 +0.15287 -0.09817
[...]
```

Contrast files are defined to capture main effects of threat and deprivation as well as interaction effects:

Effect of Threat:

```
0 0 0 0 1 0 0 0
```

Effect of Deprivation:

```
0 0 0 0 0 1 0 0
```

Threat interacting with age:

```
0 0 0 0 0 0 1 0
```

Deprivation interacting with age:

```
0 0 0 0 0 0 0 1
```

2.3.4 General Commands for Analysis

First we pre-process with a 15 mm smoothing kernel (for area models, area.fwhm15.fsaverage is substituted):

```
mris_preproc --fsgd subjlist.fsgd \
    --cache-in thickness.fwhm15.fsaverage \ # 15 mm smoothing kernel.
    --target fsaverage \
    --hemi lh \
    --out lh.thickness.preproc.15.mgh
```

The final models are fit using mri_glmfit (‘v 1.241.2.4 2016/12/08 22:02:40 zkaufman Exp’), eg:

```
mri_glmfit --y lh.thickness.preproc.15.mgh \
    --X threatdep.X \ # Specify the model
    --C threatdep_c1.mtx --C threatdep_c2.mtx \ # Specify the contrasts
    --surf fsaverage lh \
    --cortex \
    --glmdir lh.threatdep.thickness.15.glmdir \
    --eres-save
```

Then we cluster correct using mri_glmfit-sim. Following recommendations in Greve & Fischl (2018), we tested for significant clusters using permutation testing. This approach avoids false positives from parametric approaches which erroneously assume a Gaussian distribution of noise in the brain. Since our design is non-orthogonal, we use a new version of mri_glmfit-sim (‘mri_glmfit-sim,v 1.66 2016/11/16 20:52:22 greve Exp’) to implement the Ter Braak approximation, again as suggested by Greve & Fischl. Cluster correction is performed using the following code:

```
mri_glmfit-sim \
    --glmdir lh.threatdep.thickness.15.glmdir \
    --sim perm 2500 1.3 neg \ # Vertex-wise cluster threshold of 1.3 (log10(.05))
    --cwp 0.05\               # Cluster-wise p-value threshold
    --sim-sign neg \          # Direction of hypothesized change
    --2spaces                 # Bonferroni correction for both hemispheres
```

The resulting overlays are used in whole brain figures. Thresholds thresholds used in figure generation are 1.3 - 4, which correspond to p=.05 and p=.0001 using Freesurfer's log10 notation.

2.4 Subcortical Models

The below code estimates the models used for subcortical analyses. Left and right volumes are summed (see methods). Lavaan can generate warnings when variables at very different scales are entered in to the same model, so we also divide ICV and roi volumes by constants.

```
df$ICV<-df$EstimatedTotalIntraCranialVol/100000
df$Hippocampus<-df$Hippocampus.orig/1000

m.threat.hipp<-sem("Hippocampus~threat+S3AGE+FEMALE+ICV+logINR",
                data=df,
                missing='fiml',
                fixed.x=FALSE)
m.dep.hipp<-sem("Hippocampus~dep+S3AGE+FEMALE+ICV+logINR",
                data=df,
                missing='fiml',
                fixed.x=FALSE)
m.threatdep.hipp<-sem("Hippocampus~dep+threat+S3AGE+FEMALE+ICV+logINR",
                data=df,
                missing='fiml',
                fixed.x=FALSE)

df$Amygdala<-df$Amygdala.orig/1000
m.threat.amyg<-sem("Amygdala~threat+S3AGE+FEMALE+ICV+logINR",
                data=df,
                missing='fiml',
                fixed.x=FALSE)
m.dep.amyg<-sem("Amygdala~dep+S3AGE+FEMALE+ICV+logINR",
                data=df,
                missing='fiml',
                fixed.x=FALSE)
m.threatdep.amyg<-sem("Amygdala~dep+threat+S3AGE+FEMALE+ICV+logINR",
                data=df,
                missing='fiml',
                fixed.x=FALSE)
```

2.4.1 Subcortical Age Interaction

```
m.threatdep.hipp.ageint <-
    sem(
```

```r
        "Hippocampus~dep+threat:S3AGE.c+threat+S3AGE.c+FEMALE+ICV+logINR",
        data = df,
        missing = 'fiml',
        fixed.x = FALSE
    )

m.threatdep.amyg.ageint <-
    sem(
        "Amygdala~dep+threat:S3AGE.c+threat+S3AGE.c+FEMALE+ICV+logINR",
        data = df,
        missing = 'fiml',
        fixed.x = FALSE
    )

m.threatdep.amyg.ageint.null <-
    sem(
        "Amygdala~dep+0*threat:S3AGE.c+threat+S3AGE.c+FEMALE+ICV+logINR",
        data = df,
        missing = 'fiml',
        fixed.x = FALSE
    )

summary(compareFit(m.threatdep.amyg.ageint.null,m.threatdep.amyg.ageint))
```

```
## ################### Nested Model Comparison ########################
## Chi-Squared Difference Test
##
##                                Df    AIC    BIC  Chisq Chisq diff Df diff
## m.threatdep.amyg.ageint         0 4252.5 4388.1 0.0000
## m.threatdep.amyg.ageint.null    1 4255.7 4388.2 5.1116     5.1116       1
##                                Pr(>Chisq)
## m.threatdep.amyg.ageint
## m.threatdep.amyg.ageint.null    0.02377 *
## ---
## Signif. codes:  0 '***' 0.001 '**' 0.01 '*' 0.05 '.' 0.1 ' ' 1
##
## ####################### Model Fit Indices ###########################
##                                 chisq df pvalue rmsea    cfi    tli  srmr
## m.threatdep.amyg.ageint         .000†        NA .000† 1.000† 1.000† .000†
## m.threatdep.amyg.ageint.null 5.112   1   .024 .160   .929   .503   .018
##                                     aic       bic
## m.threatdep.amyg.ageint      4252.539† 4388.120†
## m.threatdep.amyg.ageint.null 4255.650  4388.151
##
## ################## Differences in Fit Indices #######################
##                                                       df rmsea    cfi    tli
## m.threatdep.amyg.ageint.null - m.threatdep.amyg.ageint  1  0.16 -0.071 -0.497
##                                                        srmr   aic  bic
## m.threatdep.amyg.ageint.null - m.threatdep.amyg.ageint 0.018 3.112 0.03
```

Age × Threat is marginal for Hippocampal volume (p=.056) and significant for Amygdala volume (p=.027). Every fit index of the interaction model is superior, although some differences are marginal (e.g. AIC).

2.4.2 Table 4

Table 4 (model summary) is then generated from the threat + deprivation model (Hippocampus) and the age interaction model (Amygdala)

```
modelsumtablecols <-
    c("rhs", "est.std", "se", "ci.lower", "ci.upper", "pvalue")
modelsumtable <-
    rbind(
        standardizedSolution(m.threatdep.hipp)[c(29, 1:6), modelsumtablecols],
        standardizedSolution(m.threatdep.amyg.ageint)[c(37, 1:7), modelsumtablecols]
    )
# starPs from https://github.com/mrpeverill/sdlabFunctions
modelsumtable$stars<-starPs(modelsumtable$pvalue)
#write.csv(modelsumtable[,c(1,2,7,3:6)],file="table-modelsummary.csv")
```

2.5 Figure generation code

2.5.1 Figure 1 Exposure Scores

```
library(ggExtra)
df$threatz<-scale(df$threat)
df$depz<-scale(df$dep)

p <- ggplot(df, aes(x=threatz, y=depz)) +
    theme_bw() +
    labs(x="Threat (z)",y="Deprivation (z)") +
    theme(panel.grid.major = element_blank(), panel.grid.minor = element_blank()) +
    geom_jitter(width = 0, height = .2) +
    theme(legend.position="none")

#ggsave("figure-exposurescatter.svg",p,width=3.25,height=4,units="in")
```

2.5.2 Figure 2 Whole Brain Analysis

Figure 2 was composed in a vector graphics program using parametric maps generated by our whole brain analysis and superimposed on Freesurfer's fsaverage (inflated) brain surface. To generate the concordance diagram, a custom script was used to generate an annotation file based on binarized masks calculated from the component overlays.

2.5.3 Figure 3 Subcortical Analysis

Figure 3 depicts the interaction effect of age and threat composite on bilateral amygdala volume. The function 'interactive' is adapted from the web application interActive (see McCabe, et. al. 2018).

```
amyginteraction <-
    interactive(
        df = df,
        i_cat = FALSE,
        i_foc = "threat",
```

```
        i_mod = "S3AGE",
        i_dv = "Amygdala.orig",
        i_xaxislab = "Threat Composite Score",
        i_yaxislab = "Amygdala Volume",
        i_title = "",
        i_covars = c("dep","ICV","FEMALE"),
        i_greyscale = TRUE,
        i_sm1=-1.6,
        i_sm2=-.8,
        i_sm3=.0,
        i_sm4=.8,
        i_sm5=1.6
    )

amyginteraction.plot<-amyginteraction$plotfinal + ylim(2500,5500)
# ggsave(
#   "figure-amygageinteraction.svg",
#   device = svg,
#   amyginteraction.plot,
#   height = 4,
#   width = 7.5
# )
```

The cut point labels are manually edited to correspond to years of age (rather than age z-score) in the final plot.

3 Supplemental Analyses

3.1 Sub-cortical Laterality Tests

Previous studies have found effects of early life maltreatment on subcortical structure which were specific to one hemisphere, however such reports are heterogeneous. Here we present models of sub-cortical effects specific to left and right hemisphere.

3.1.1 Main Effects

```
df$Left.Hippocampus.s<-df$Left.Hippocampus/1000
df$Right.Hippocampus.s<-df$Right.Hippocampus/1000

m.threat.hipp.lh<-sem("Left.Hippocampus.s~threat+S3AGE+FEMALE+ICV+logINR",
                data=df,
                missing='fiml',
                fixed.x=FALSE)
m.dep.hipp.lh<-sem("Left.Hippocampus.s~dep+S3AGE+FEMALE+ICV+logINR",
                data=df,
                missing='fiml',
                fixed.x=FALSE)
m.threatdep.hipp.lh<-sem("Left.Hippocampus.s~dep+threat+S3AGE+FEMALE+ICV+logINR",
                data=df,
                missing='fiml',
```

```
                fixed.x=FALSE)

m.threat.hipp.rh<-sem("Right.Hippocampus.s~threat+S3AGE+FEMALE+ICV+logINR",
                data=df,
                missing='fiml',
                fixed.x=FALSE)
m.dep.hipp.rh<-sem("Right.Hippocampus.s~dep+S3AGE+FEMALE+ICV+logINR",
                data=df,
                missing='fiml',
                fixed.x=FALSE)
m.threatdep.hipp.rh<-sem("Right.Hippocampus.s~dep+threat+S3AGE+FEMALE+ICV+logINR",
                data=df,
                missing='fiml',
                fixed.x=FALSE)

df$Left.Amygdala.s<-df$Left.Amygdala/1000
df$Right.Amygdala.s<-df$Right.Amygdala/1000

m.threat.amyg.lh<-sem("Left.Amygdala.s~threat+S3AGE+FEMALE+ICV+logINR",
                data=df,
                missing='fiml',
                fixed.x=FALSE)
m.dep.amyg.lh<-sem("Left.Amygdala.s~dep+S3AGE+FEMALE+ICV+logINR",
                data=df,
                missing='fiml',
                fixed.x=FALSE)
m.threatdep.amyg.lh<-sem("Left.Amygdala.s~dep+threat+S3AGE+FEMALE+ICV+logINR",
                data=df,
                missing='fiml',
                fixed.x=FALSE)

m.threat.amyg.rh<-sem("Right.Amygdala.s~threat+S3AGE+FEMALE+ICV+logINR",
                data=df,
                missing='fiml',
                fixed.x=FALSE)
m.dep.amyg.rh<-sem("Right.Amygdala.s~dep+S3AGE+FEMALE+ICV+logINR",
                data=df,
                missing='fiml',
                fixed.x=FALSE)
m.threatdep.amyg.rh<-sem("Right.Amygdala.s~dep+threat+S3AGE+FEMALE+ICV+logINR",
                data=df,
                missing='fiml',
                fixed.x=FALSE)

hipplattable <- cbind(
    Model = c(
        "Left Hippocampus",
        "Right Hippocampus",
        "Left Hippocampus",
        "Right Hippocampus",
        "Left Hippocampus (dual exp.)",
        "",
        "Right Hippocampus (dual exp.)",
```

```
        ""
    ),
    rbind(
        standardizedSolution(m.threat.hipp.lh)[1, modelsumtablecols],
        standardizedSolution(m.threat.hipp.rh)[1, modelsumtablecols],
        standardizedSolution(m.dep.hipp.lh)[1, modelsumtablecols],
        standardizedSolution(m.dep.hipp.rh)[1, modelsumtablecols],
        standardizedSolution(m.threatdep.hipp.lh)[1:2, modelsumtablecols],
        standardizedSolution(m.threatdep.hipp.rh)[1:2, modelsumtablecols]
    )
)

amyglattable <- cbind(
    Model = c(
        "Left Amygdala",
        "Right Amygdala",
        "Left Amygdala",
        "Right Amygdala",
        "Left Amygdala (dual exp.)",
        "",
        "Right Amygdala (dual exp.)",
        ""
    ),
    rbind(
        standardizedSolution(m.threat.amyg.lh)[1, modelsumtablecols],
        standardizedSolution(m.threat.amyg.rh)[1, modelsumtablecols],
        standardizedSolution(m.dep.amyg.lh)[1, modelsumtablecols],
        standardizedSolution(m.dep.amyg.rh)[1, modelsumtablecols],
        standardizedSolution(m.threatdep.amyg.lh)[1:2, modelsumtablecols],
        standardizedSolution(m.threatdep.amyg.rh)[1:2, modelsumtablecols]
    )
)
```

Hippocampus volume was not related to any exposure in any model.

Table S5: Hippocampal models (lateralized); exposure terms

Model	rhs	est.std	se	ci.lower	ci.upper	pvalue
Left Hippocampus	threat	-0.0961071	0.0780769	-0.2491349	0.0569208	0.2183494
Right Hippocampus	threat	-0.0065731	0.0794341	-0.1622612	0.1491149	0.9340508
Left Hippocampus	dep	0.0147594	0.0771873	-0.1365250	0.1660438	0.8483571
Right Hippocampus	dep	-0.0029272	0.0779813	-0.1557678	0.1499133	0.9700563
Left Hippocampus (dual exp.)	dep	0.0996661	0.0938960	-0.0843666	0.2836987	0.2884845
	threat	-0.1517265	0.0957954	-0.3394820	0.0360290	0.1132255
Right Hippocampus (dual exp.)	dep	-0.0023539	0.0963662	-0.1912283	0.1865204	0.9805120
	threat	-0.0022219	0.0985292	-0.1953356	0.1908918	0.9820088

Threat was related to Amygdala volume in right hemisphere and marginally significant in the left, before control for deprivation. After control for deprivation, threat was related to Amygdala volume in left hemisphere and was marginally significant in the right.

Deprivation was not related to Amygdala volume in any model.

Table S6: Amygdala models (lateralized); exposure terms

Model	rhs	est.std	se	ci.lower	ci.upper	pvalue
Left Amygdala	threat	-0.1593620	0.0826778	-0.3214075	0.0026835	0.0539165
Right Amygdala	threat	-0.1855079	0.0816896	-0.3456166	-0.0253993	0.0231537
Left Amygdala	dep	-0.0163201	0.0824078	-0.1778364	0.1451961	0.8430130
Right Amygdala	dep	-0.0970186	0.0816373	-0.2570247	0.0629876	0.2346720
Left Amygdala (dual exp.)	dep	0.1197829	0.0997742	-0.0757709	0.3153367	0.2299296
	threat	-0.2333722	0.1016685	-0.4326388	-0.0341057	0.0217089
Right Amygdala (dual exp.)	dep	0.0189615	0.0999194	-0.1768770	0.2147999	0.8494911
	threat	-0.1960337	0.1017726	-0.3955043	0.0034369	0.0540802

3.1.2 Age Interaction

```
m.threatdep.hipp.ageint.lh <- sem(
    "Left.Hippocampus.s~dep+threat:S3AGE.c+threat+S3AGE.c+FEMALE+ICV+logINR",
    data = df,
    missing = 'fiml',
    fixed.x = FALSE
)
pander(standardizedsolution(m.threatdep.hipp.ageint.lh)[1:7,])
```

lhs	op	rhs	est.std	se	z	pvalue	ci.lower	ci.upper
Left.Hippocampus.s	~	dep	0.089	0.093	0.960	0.337	-0.093	0.272
Left.Hippocampus.s	~	threat:S3AGE.c	0.126	0.077	1.640	0.101	-0.025	0.276
Left.Hippocampus.s	~	threat	-0.159	0.095	-1.671	0.095	-0.345	0.027
Left.Hippocampus.s	~	S3AGE.c	-0.187	0.082	-2.279	0.023	-0.348	-0.026
Left.Hippocampus.s	~	FEMALE	-0.022	0.081	-0.272	0.786	-0.182	0.137
Left.Hippocampus.s	~	ICV	0.565	0.076	7.384	0.000	0.415	0.715
Left.Hippocampus.s	~	logINR	0.096	0.085	1.120	0.263	-0.072	0.263

```
m.threatdep.hipp.ageint.rh <- sem(
    "Right.Hippocampus.s~dep+threat:S3AGE.c+threat+S3AGE.c+FEMALE+ICV+logINR",
    data = df,
    missing = 'fiml',
    fixed.x = FALSE
)
pander(standardizedsolution(m.threatdep.hipp.ageint.rh)[1:7, ])
```

lhs	op	rhs	est.std	se	z	pvalue	ci.lower	ci.upper
Right.Hippocampus.s	~	dep	-0.014	0.095	-0.150	0.881	-0.201	0.173
Right.Hippocampus.s	~	threat:S3AGE.c	0.152	0.078	1.940	0.052	-0.002	0.306
Right.Hippocampus.s	~	threat	-0.011	0.097	-0.109	0.913	-0.201	0.180
Right.Hippocampus.s	~	S3AGE.c	-0.113	0.084	-1.347	0.178	-0.278	0.052
Right.Hippocampus.s	~	FEMALE	-0.062	0.083	-0.752	0.452	-0.225	0.100
Right.Hippocampus.s	~	ICV	0.539	0.078	6.864	0.000	0.385	0.693
Right.Hippocampus.s	~	logINR	0.102	0.085	1.195	0.232	-0.065	0.269

```r
m.threatdep.amyg.ageint.lh <- sem(
    "Left.Amygdala.s~dep+threat:S3AGE.c+threat+S3AGE.c+FEMALE+ICV+logINR",
    data = df,
    missing = 'fiml',
    fixed.x = FALSE
)
pander(standardizedsolution(m.threatdep.amyg.ageint.lh)[1:7, ])
```

lhs	op	rhs	est.std	se	z	pvalue	ci.lower	ci.upper
Left.Amygdala.s	~	dep	0.106	0.099	1.080	0.280	-0.087	0.300
Left.Amygdala.s	~	threat:S3AGE.c	0.165	0.081	2.036	0.042	0.006	0.325
Left.Amygdala.s	~	threat	-0.242	0.100	-2.419	0.016	-0.439	-0.046
Left.Amygdala.s	~	S3AGE.c	-0.033	0.087	-0.377	0.706	-0.204	0.139
Left.Amygdala.s	~	FEMALE	-0.088	0.086	-1.017	0.309	-0.256	0.081
Left.Amygdala.s	~	ICV	0.416	0.085	4.917	0.000	0.250	0.582
Left.Amygdala.s	~	logINR	0.032	0.089	0.358	0.720	-0.143	0.207

```r
m.threatdep.amyg.ageint.rh <- sem(
    "Right.Amygdala.s~dep+threat:S3AGE.c+threat+S3AGE.c+FEMALE+ICV+logINR",
    data = df,
    missing = 'fiml',
    fixed.x = FALSE
)
pander(standardizedsolution(m.threatdep.amyg.ageint.rh)[1:7, ])
```

lhs	op	rhs	est.std	se	z	pvalue	ci.lower	ci.upper
Right.Amygdala.s	~	dep	0.004	0.098	0.045	0.964	-0.189	0.197
Right.Amygdala.s	~	threat:S3AGE.c	0.174	0.081	2.153	0.031	0.016	0.333
Right.Amygdala.s	~	threat	-0.207	0.100	-2.062	0.039	-0.403	-0.010
Right.Amygdala.s	~	S3AGE.c	-0.055	0.087	-0.635	0.526	-0.226	0.116
Right.Amygdala.s	~	FEMALE	-0.197	0.085	-2.307	0.021	-0.364	-0.030
Right.Amygdala.s	~	ICV	0.369	0.085	4.326	0.000	0.202	0.537
Right.Amygdala.s	~	logINR	-0.105	0.089	-1.175	0.240	-0.279	0.070

In the fully specified age interaction model, effects of threat on Amygdala volume were slightly stronger in the right hemisphere than in the left, but remained significant in the left hemisphere.

3.2 Pubertal Timing Interaction Analysis

Pubertal stage was determined using self-report tanner staging, which is correlated to physicians' physical examinations of pubertal development. For further details on pubertal timing assessment and association with early life adversity in the larger study from which the present sample was drawn, see Sumner, et. al. (2019).

Pubertal timing was missing at higher rates than other variables in the study.

```r
df$TANNER_MISS<-is.na(df$TANNER_STAGE)
pander(summary(lm(TANNER_MISS ~ S3AGE.c + FEMALE + threat + dep,data=df)))
```

	Estimate	Std. Error	t value	Pr(>|t|)
**(Intercept)**	0.147	0.041	3.617	0.000
**S3AGE.c**	-0.024	0.010	-2.470	0.015
**FEMALE**	-0.033	0.051	-0.658	0.511
**threat**	-0.010	0.016	-0.602	0.548
**dep**	0.047	0.035	1.341	0.182

Table S12: Fitting linear model: TANNER_MISS ~ S3AGE.c + FEMALE + threat + dep

Observations	Residual Std. Error	R^2	Adjusted R^2
161	0.319	0.053	0.029

For use in whole brain models, missing data from 19 participants was imputed based on participants age (see Figure S3).

```
m.tannerpred<-lm(TANNER_STAGE ~ S3AGE.c,data=df)
df$TANNER_PRED<-predict(m.tannerpred,df)
df$TANNER_IMPUTED<-df$TANNER_PRED
df[!df$TANNER_MISS,"TANNER_IMPUTED"]<-df[!df$TANNER_MISS,"TANNER_STAGE"]

ggplot(df,aes(y=TANNER_IMPUTED,x=S3AGE,shape=TANNER_MISS)) +
    geom_point(size=1.5) +
    scale_shape_manual(values=c(16, 1)) +
    labs(x="Age",
         y="Tanner Score with Imputations",
         shape="Imputed")
```

Whole brain models were run as in the age-interaction analysis, above, with the (centered) tanner_stage variable used instead of age and when calculating interaction effects with threat and deprivation. No significant clusters of thinning were observed.

Our sub-cortical models handle missing data using FIML, which is superior to the imputation described above.

```
df$Tanner<-df$TANNER_STAGE #Abbreviation for legibility
m.threatdep.amyg.pubint<-sem(
    "Amygdala~dep+threat:Tanner+threat+Tanner+FEMALE+ICV+logINR",
  data=df,
  missing='fiml',
  fixed.x=FALSE)
pander(standardizedsolution(m.threatdep.amyg.pubint)[1:7,])
```

lhs	op	rhs	est.std	se	z	pvalue	ci.lower	ci.upper
Amygdala	~	dep	0.074	0.097	0.769	0.442	-0.115	0.264
Amygdala	~	threat:Tanner	0.298	0.181	1.643	0.100	-0.057	0.653
Amygdala	~	threat	-0.481	0.187	-2.571	0.010	-0.848	-0.114
Amygdala	~	Tanner	-0.074	0.086	-0.855	0.393	-0.242	0.095
Amygdala	~	FEMALE	-0.135	0.085	-1.583	0.113	-0.301	0.032

lhs	op	rhs	est.std	se	z	pvalue	ci.lower	ci.upper
Amygdala	~	ICV	0.424	0.085	4.976	0.000	0.257	0.591
Amygdala	~	logINR	-0.031	0.088	-0.352	0.725	-0.203	0.141

No interaction between threat and pubertal stage was found.

3.3 Main effect of exposure on subcortical volumes.

Figure S4 shows main effects of exposure composites on Amygdala and Hippocampus volume.

```
roiplot <- list(geom_point(),
                geom_smooth(method = 'lm', formula = y ~ x))

roiplot2 <- list(geom_jitter(width = .2, height = 0),
                 geom_smooth(method = 'lm', formula = y ~ x))

amygdthreat <- ggplot(df, aes(x = threatz, y = Amygdala)) +
  labs(title = "Amygdala Volume",
       y =  "Volume (mm^3)",
       x = "Threat Experiences") +
  roiplot + ylim(1390, 2800) +
  annotate("text",
           x = .5,
           y = (2800 - 1390) * .95 + 1390,
           label = sprintf("  = %.2f, p = %.3f*",
                           standardizedSolution(m.threat.amyg)[1, 4],
                           standardizedSolution(m.threat.amyg)[1, 7]),
           family = theme_get()$text[["family"]],
           size = theme_get()$text[["size"]] / 2.5)

amygddep <- ggplot(df, aes(x = depz, y = Amygdala)) +
  labs(title = "Amygdala Volume",
       y = "Volume (mm^3)",
       x = "Deprivation Experiences") +
  roiplot2 + ylim(1390, 2800) +
  annotate("text",
           x = 1,
           y = (2800 - 1390) * .95 + 1390,
           label = sprintf("  = %.2f, p = %.3f",
                           standardizedSolution(m.dep.amyg)[1, 4],
                           standardizedSolution(m.dep.amyg)[1, 7]),
           family = theme_get()$text[["family"]],
           size = theme_get()$text[["size"]] / 2.5)

hippthreat <-  ggplot(df, aes(x = threatz, y = Hippocampus)) +
  labs(title = "Hippocampal Volume",
       y = "Volume (mm^3)", x = "Threat Experiences") +
  roiplot + ylim(3795, 6300) +
  annotate("text",
           x = .5,
           y = (6300 - 3795) * .95 + 3795,
           label = sprintf("  = %.2f, p = %.3f",
```

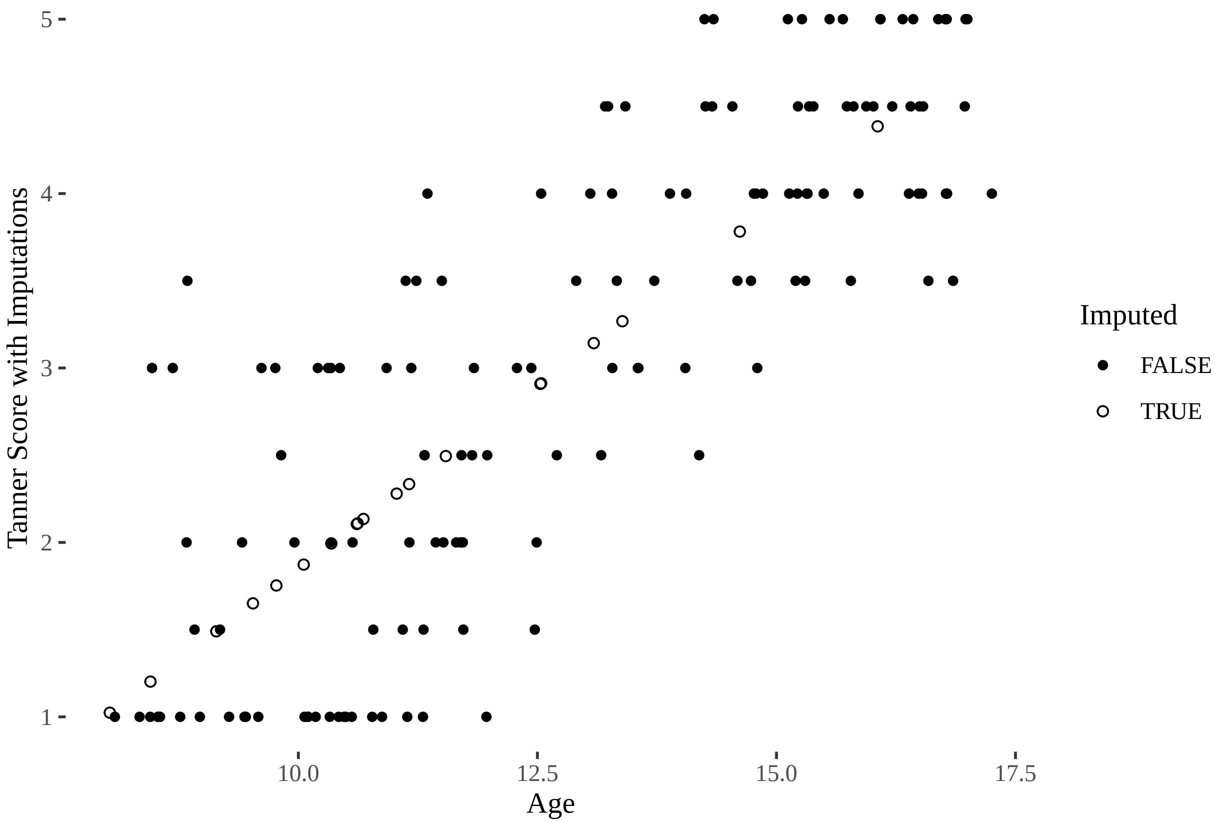

Figure S3: Tanner Score and Age with Imputations

```
                            standardizedSolution(m.threat.hipp)[1, 4],
                            standardizedSolution(m.threat.hipp)[1, 7]),
          family = theme_get()$text[["family"]],
          size = theme_get()$text[["size"]] / 2.5)

hippdep <- ggplot(df, aes(x = depz, y = Hippocampus)) +
  labs(title = "Hippocampal Volume",
       y = "Volume (mm^3)",
       x = "Deprivation Experiences") +
  roiplot2 + ylim(3795, 6300) +
  annotate("text",
          x = 1,
          y = (6300 - 3795) * .95 + 3795,
          label = sprintf("  = %.2f, p = %.3f",
                          standardizedSolution(m.dep.hipp)[1, 4],
                          standardizedSolution(m.dep.hipp)[1, 7]),
          family = theme_get()$text[["family"]],
          size = theme_get()$text[["size"]] / 2.5)

#to save the plot:
#svg(filename="figure-subcortanalysis.svg",width=7.5,height=5)
#(amygdthreat + amygddep) / (hippthreat + hippdep)
#dev.off()
```

Minor legibility edits were made to the resulting figure in a vector graphics program (see Figure S4).

3.4 Age × Threat Interaction on Hippocampal Volume

The threat × age term from the Hippocampal Volume age interaction model was non-significant (p=.056)

Table S14: Hippocampal threat × age interaction model; standardized estimates

	rhs	est.std	se	ci.lower	ci.upper	pvalue	stars
29	ICV	0.2156159	0.0766164	0.0654506	0.3657812	0.0048895	**
1	dep	0.0402176	0.0921326	-0.1403589	0.2207941	0.6624602	
2	threat:S3AGE.c	0.1447192	0.0757679	-0.0037832	0.2932216	0.0561290	
3	threat	-0.0896532	0.0939724	-0.2738357	0.0945293	0.3400646	
4	S3AGE.c	-0.1574308	0.0812551	-0.3166879	0.0018262	0.0526856	
5	FEMALE	-0.0436880	0.0802222	-0.2009206	0.1135446	0.5860372	
6	ICV	0.5755976	0.0750472	0.4285078	0.7226874	0.0000000	***
7	logINR	0.1030702	0.0834222	-0.0604342	0.2665746	0.2166349	

Figure S5 shows an estimate of the relation of hippocampal volume with threat score across levels of age.

```
hippinteraction <-
    interactive(
        df = df,
        i_cat = FALSE,
        i_foc = "threat",
        i_mod = "S3AGE",
```

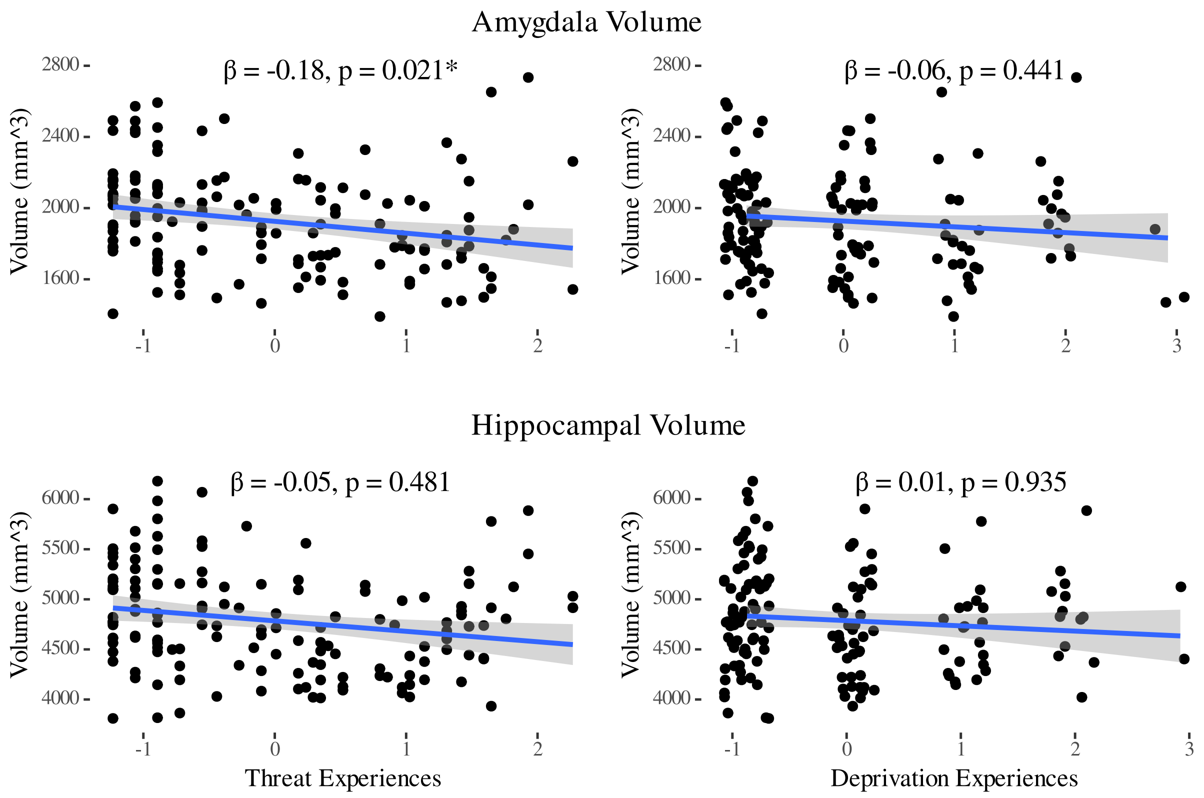

Figure S4: Threat and deprivation plotted against bilateral Amygdala and Hippocampus volume (Deprivation trend lines non-significant)

```
        i_dv = "Hippocampus.orig",
        i_xaxislab = "Threat Composite Score",
        i_yaxislab = "Hippocampus Volume",
        i_title = "",
        i_covars = c("dep","ICV","FEMALE"),
        i_greyscale = TRUE,
        i_sm1=-1.6,
        i_sm2=-.8,
        i_sm3=.0,
        i_sm4=.8,
        i_sm5=1.6
    )

hippinteraction.plot<-hippinteraction$plotfinal + ylim(7500,13000)
```

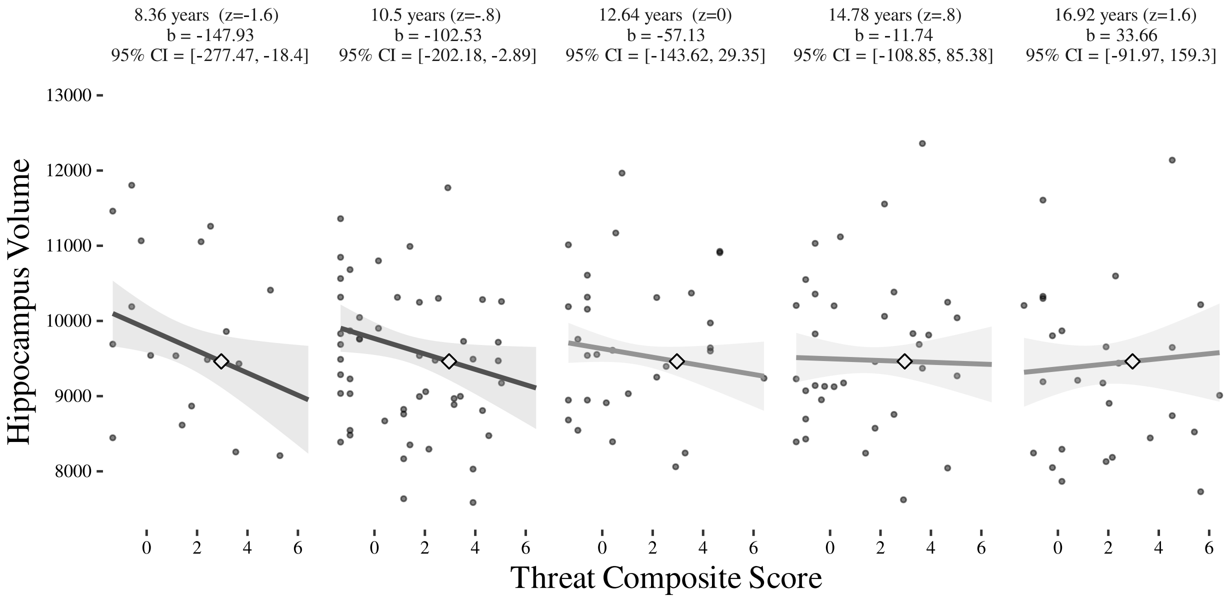

Figure S5: Marginal interaction of threat and age on Hippocampal volume

4 R Environment

R packages used were as follows:

R version 4.2.1 (2022-06-23)

Platform: x86_64-pc-linux-gnu (64-bit)

locale: *LC_CTYPE=C.UTF-8*, *LC_NUMERIC=C*, *LC_TIME=C.UTF-8*, *LC_COLLATE=C.UTF-8*, *LC_MONETARY=C.UTF-8*, *LC_MESSAGES=C.UTF-8*, *LC_PAPER=C.UTF-8*, *LC_NAME=C*, *LC_ADDRESS=C*, *LC_TELEPHONE=C*, *LC_MEASUREMENT=C.UTF-8* and *LC_IDENTIFICATION=C*

attached base packages: *stats*, *graphics*, *grDevices*, *utils*, *datasets*, *methods* and *base*

other attached packages: *QuantPsyc(v.1.6)*, *MASS(v.7.3-57)*, *boot(v.1.3-28)*, *RColorBrewer(v.1.1-3)*, *ggExtra(v.0.10.0)*, *semTools(v.0.5-6)*, *forcats(v.0.5.1)*, *stringr(v.1.4.0)*, *dplyr(v.1.0.9)*, *purrr(v.0.3.4)*, *readr(v.2.1.2)*, *tidyr(v.1.2.0)*, *tibble(v.3.1.7)*, *tidyverse(v.1.3.1)*, *lsr(v.0.5.2)*, *reshape2(v.1.4.4)*, *ggthemes(v.4.2.4)*, *car(v.3.1-0)*, *carData(v.3.0-5)*, *patchwork(v.1.1.1)*, *see(v.0.7.2)*, *lavaan(v.0.6-12)*, *tables(v.0.9.6)*, *sdlabFunctions(v.0.2.0)*, *pander(v.0.6.5)*, *summarytools(v.1.0.1)*, *Hmisc(v.4.7-0)*, *ggplot2(v.3.3.6)*, *Formula(v.1.2-4)*, *survival(v.3.2-13)* and *lattice(v.0.20-45)*

loaded via a namespace (and not attached): *colorspace(v.2.0-3)*, *deldir(v.1.0-6)*, *pryr(v.0.1.5)*, *ellipsis(v.0.3.2)*, *estimability(v.1.4.1)*, *htmlTable(v.2.4.1)*, *base64enc(v.0.1-3)*, *fs(v.1.5.2)*, *rstudioapi(v.0.13)*, *farver(v.2.1.1)*, *fansi(v.1.0.3)*, *mvtnorm(v.1.1-3)*, *lubridate(v.1.8.0)*, *xml2(v.1.3.3)*, *codetools(v.0.2-18)*, *splines(v.4.2.1)*, *mnormt(v.2.1.0)*, *knitr(v.1.39)*, *jsonlite(v.1.8.0)*, *broom(v.1.0.0)*, *cluster(v.2.1.3)*, *dbplyr(v.2.2.1)*, *png(v.0.1-7)*, *shiny(v.1.7.1)*, *compiler(v.4.2.1)*, *httr(v.1.4.3)*, *emmeans(v.1.8.0)*, *backports(v.1.4.1)*, *assertthat(v.0.2.1)*, *Matrix(v.1.4-1)*, *fastmap(v.1.1.0)*, *cli(v.3.3.0)*, *later(v.1.3.0)*, *htmltools(v.0.5.2)*, *tools(v.4.2.1)*, *coda(v.0.19-4)*, *gtable(v.0.3.0)*, *glue(v.1.6.2)*, *Rcpp(v.1.0.9)*, *cellranger(v.1.1.0)*, *vctrs(v.0.4.1)*, *xfun(v.0.31)*, *rvest(v.1.0.2)*, *mime(v.0.12)*, *miniUI(v.0.1.1.1)*, *lifecycle(v.1.0.1)*, *scales(v.1.2.0)*, *promises(v.1.2.0.1)*, *hms(v.1.1.1)*, *parallel(v.4.2.1)*, *yaml(v.2.3.5)*, *gridExtra(v.2.3)*, *rpart(v.4.1.16)*, *latticeExtra(v.0.6-30)*, *stringi(v.1.7.6)*, *highr(v.0.9)*, *checkmate(v.2.1.0)*, *rlang(v.1.0.3)*, *pkgconfig(v.2.0.3)*, *matrixStats(v.0.62.0)*, *evaluate(v.0.15)*, *labeling(v.0.4.2)*, *rapportools(v.1.1)*, *htmlwidgets(v.1.5.4)*, *tidyselect(v.1.1.2)*, *plyr(v.1.8.7)*, *magrittr(v.2.0.3)*, *R6(v.2.5.1)*, *magick(v.2.7.3)*, *generics(v.0.1.3)*, *DBI(v.1.1.3)*, *pillar(v.1.7.0)*, *haven(v.2.5.0)*, *foreign(v.0.8-82)*, *withr(v.2.5.0)*, *abind(v.1.4-5)*, *nnet(v.7.3-17)*, *modelr(v.0.1.8)*, *crayon(v.1.5.1)*, *interp(v.1.1-3)*, *utf8(v.1.2.2)*, *tzdb(v.0.3.0)*, *rmarkdown(v.2.14)*, *jpeg(v.0.1-9)*, *grid(v.4.2.1)*, *readxl(v.1.4.0)*, *data.table(v.1.14.2)*, *pbivnorm(v.0.6.0)*, *reprex(v.2.0.1)*, *digest(v.0.6.29)*, *xtable(v.1.8-4)*, *httpuv(v.1.6.5)*, *stats4(v.4.2.1)*, *munsell(v.0.5.0)* and *tcltk(v.4.2.1)*

5 Supplemental References

Baldwin, J. R., Reuben, A., Newbury, J. B., & Danese, A. (2019). Agreement Between Prospective and Retrospective Measures of Childhood Maltreatment: A Systematic Review and Meta-analysis. *JAMA Psychiatry*, 76(6), 584. https://doi.org/10.1001/jamapsychiatry.2019.0097

Greve, D. N., & Fischl, B. (2018). False positive rates in surface-based anatomical analysis. *NeuroImage*, 171, 6–14. https://doi.org/10.1016/j.neuroimage.2017.12.072

Hardt, J., & Rutter, M. (2004). Validity of adult retrospective reports of adverse childhood experiences: Review of the evidence. *Journal of Child Psychology and Psychiatry*, 45(2), 260–273. https://doi.org/10.1111/j.1469-7610.2004.00218.x

McCabe, C. J., Kim, D. S., & King, K. M. (2018). Improving Present Practices in the Visual Display of Interactions. *Advances in Methods and Practices in Psychological Science*, 147–165.

Sumner, J. A., Colich, N. L., Uddin, M., Armstrong, D., & McLaughlin, K. A. (2019). Early experiences of threat, but not deprivation, are associated with accelerated biological aging in children and adolescents. *Biological Psychiatry*, 85(3), 268–278. https://doi.org/10.1016/j.biopsych.2018.09.008

Widom, C. S., Raphael, K. G., & DuMont, K. A. (2004). The case for prospective longitudinal studies in child maltreatment research: Commentary on Dube, Williamson, Thompson, Felitti, and Anda (2004). *Child Abuse & Neglect*, 28(7), 715–722. https://doi.org/10.1016/j.chiabu.2004.03.009
